# Prospects for Creation of Cardioprotective and Antiarrhythmic Drugs Based on Opioid Receptor Agonists

**DOI:** 10.1002/med.21395

**Published:** 2016-05-16

**Authors:** Leonid N Maslov, Igor Khaliulin, Peter R. Oeltgen, Natalia V. Naryzhnaya, Jian‐Ming Pei, Stephen A. Brown, Yury B. Lishmanov, James M. Downey

**Affiliations:** ^1^ Research Institute for CardiologyTomskRussia; ^2^ School of Clinical SciencesUniversity of BristolBristolUK; ^3^ VA Medical Center, University of KentuckyLexingtonKY; ^4^ Department of PhysiologyFourth Military Medical UniversityXi'anP. R. China; ^5^ National Research Tomsk Polytechnic University634050TomskRussia; ^6^ University of South AlabamaMobileAL

**Keywords:** opioids, heart, ischemia/reperfusion injury, cardioprotection, signal transduction

## Abstract

It has now been demonstrated that the μ, δ_1_, δ_2_, and κ_1_ opioid receptor (OR) agonists represent the most promising group of opioids for the creation of drugs enhancing cardiac tolerance to the detrimental effects of ischemia/reperfusion (I/R). Opioids are able to prevent necrosis and apoptosis of cardiomyocytes during I/R and improve cardiac contractility in the reperfusion period. The OR agonists exert an infarct‐reducing effect with prophylactic administration and prevent reperfusion‐induced cardiomyocyte death when ischemic injury of heart has already occurred; that is, opioids can mimic preconditioning and postconditioning phenomena. Furthermore, opioids are also effective in preventing ischemia‐induced arrhythmias.

## 1. HISTORICAL BACKGROUND

The main cardiac manifestations of ischemia/reperfusion (I/R) are necrosis, apoptosis of cardiomyocytes, contractile dysfunction, and ventricular arrhythmias.^1^, ^2^, ^3^, ^4^ In the 70s it was clear that the prognosis for patients who had suffered acute myocardial infarction was highly dependent on the amount of ventricular muscle that was lost to infarction. Although it was proposed that an intervention that could reduce infarct size would save lives, there was great debate as to whether therapeutic attenuation of these negative manifestations of myocardial ischemia was even possible. This argument was settled once and for all in 1986, when three American researchers discovered the phenomenon of ischemic preconditioning (IP).^5^ They found that exposure to four brief periods of I/R causes the heart to become very resistant to infarction from a subsequent prolonged ischemic insult. The protective effect of IP is maintained only for 2–3 hr, making it impractical for any clinical application. However, a day later these hearts again became resistant to infarction, this time lasting about 4 days. This has been variously called “delayed preconditioning,” “late preconditioning,” or “second window of protection.”^6^ Still the major impediment to translating preconditioning to a clinical setting was the requirement that it has to be instituted prior to the onset of ischemia. Pretreatment is impossible, however, in the setting of acute myocardial infarction.

Although the mechanism of IP was obscure, it was strongly believed that it must target injury during ischemia and that the pretreatment requisite was absolute. It took another 17 years after the discovery of IP before it was realized that IP actually prevents reperfusion injury and treatment could be instituted right up to the time of reperfusion. In 2003, Vinten‐Johansen's group discovered “ischemic postconditioning” (IPost). It turned out that three cycles of very brief reperfusion/ischemia cycles after a prolonged ischemic insult greatly decrease the fraction of the ischemic myocardium that infarcted, often called the “infarct size‐area at risk ratio” (IS/AAR).^7^ This seminal discovery finally opened the door to clinical application but while IPost was theoretically possible in patients whose coronary thrombus was removed with angioplasty, it proved to be surprisingly awkward in many cases. A postconditioning drug would be a great improvement.

The mechanism of IP had been obscure until it was discovered that it resulted from protective signal transduction pathways triggered by Gi‐coupled plasma membrane receptors.^8^ These investigators found that adenosine was a trigger through adenosine A1 receptors but soon it was found that other Gi‐coupled receptors also participated in triggering IP's protection. In 1995, Gross's group obtained data that the infarct‐reducing effect of IP was lost after blocking opioid receptors (ORs) with naloxone^9^ and a year later they reported that they could precondition the heart with morphine.^10^ Kin et al.^11^ demonstrated that ORs were also involved in IPost when they showed that naloxone 5 min before reperfusion abolishes the infarct‐sparing effect of IPost.

IP or IPost with ischemia is impractical in the clinical settings. The foregoing studies generated enormous interest by physiologists and pharmacologists and provided the impetus for research aimed at finding pharmaceutical OR agonists that could mimic the phenomenon of IP and IPost. In this review, we evaluate the effect of various OR ligands on the necrosis and apoptosis of cardiomyocytes, myocardial stunning, and the incidence of ischemic and reperfusion arrhythmias. To aid the reader we have included Table I, which lists all of the OR agonists and antagonists discussed in this review.

**Table I:** OR Active Drugs

ARD‐353	Nonpeptide δ_1_ and δ_2_ OR agonist (does not cross the BBB)
BNTX	δ_1_ OR antagonist
Bremazocine	κ_2_ OR agonist
BRL 52537	κ OR agonist
Buprenorphine	μ and κ OR agonist
BW373U86	δ OR agonist
Carfentanil	μ OR agonist
CTOP	μ OR antagonist
DADLE	δ OR agonist
Dalargin	μ and δ OR agonist
DALDA	μ and κ OR agonist
DAMGO	μ OR agonist
Deltorphin D	δ_2_ OR agonist
Deltorphin II	δ_2_ OR agonist
Dermorphin H	μ OR agonist
[Dmt(1)] DALDA	Di‐methyl tyrosine version of DALDA‐ potent μ and К OR agonist
DPDPE	δ_1_ OR agonist
Dynorphin	κ OR agonist
Eribis peptide 94	μ and δ OR agonist
Fentanyl	μ OR agonist
FIT	δ OR agonist
FK 33‐824	Selective μ OR agonist, synthetic analogue of Met‐enkephalin
GNTI	κ OR antagonist
GR‐89696	κ_2_ OR agonist
ICI 199,441	κ OR agonist
ICI 204,448	κ OR agonist
MEAP	Met‐enkephalin‐Arg‐Phe ‐ μ, δ and κ OR agonist
Meptazinol	μ‐OR agonist and antagonist
Met‐enkephalin	μ and δ OR agonist
Methadone	μ OR agonist
Morphine	Nonselective OR agonist
Mr 2266	κ OR antagonist
MrZ 2593	Peripheral nonselective OR antagonist (does not cross BBB at 1 mg/kg)
Naloxone methiodide	Peripheral nonselective OR antagonist (does not cross BBB)
Naloxone	Nonselective OR antagonist
Naltrexone	Nonselective OR antagonist
Naltriben	δ_2_ OR antagonist
Natrindole	Highly selective δ OR‐selective antagonist
Nociceptin	ORL1 agonist
Nor‐binaltorphimine	κ OR antagonist
PD 129290	κ OR agonist
(+)‐pentazocine	Preferential σ‐OR agonist
(−)‐pentazocine	κ‐OR agonist
Quadazocine	κ_2_ OR antagonist
Remifentanil	Nonselective OR agonist
SNC‐121	Non‐peptide δ OR agonist
SNC‐80	δ OR agonist
Sufentanil	μ OR agonist
TAN‐67	δ_1_ OR agonist
Tramadol	Nonselective agonist and antagonist of ORs
U50,488	κ_1_ OR agonist (does not cross the BBB)

## 2. LOCALIZATION OF OPIOID RECEPTORS INVOLVED IN REGULATION OF HEART FUNCTION

### A. Opioid Receptors in the Central Nervous System

It is well known that all discovered mammalian opioid peptides have been isolated from brain where they are most abundant^12^ and it is not surprising that the brain and spinal cord have a high density of ORs.^13^, ^14^, ^15^, ^16^, ^17^, ^18^, ^19^, ^20^, ^21^ μ OR was discovered in the spinal cord, in the periaqueductal gray matter, nucleus accumbens, amygdala and in several thalamic nuclei.^15^, ^19^ Transcripts of μ OR were found in the prefrontal cortex, nucleus accumbens, caudate putamen, and thalamus.^20^ δ OR was identified in spinal cord,^16^ caudate putamen, nucleus accumbens, and olfactory tubercle.^18^ Transcripts of δ OR were detected in the prefrontal cortex, nucleus accumbens, and caudate putamen.^20^ The κ OR was found in spinal cord.^16^ Transcripts for this receptor were identified in nucleus accumbens, caudate putamen, preoptic area, and hypothalamus.^20^ κ OR was also found in the prefrontal cortex, nucleus accumbens, hypothalamus, amygdala, ventral tegmental area, dorsal raphe nucleus, and locus coeruleus.^21^ The ORL1 receptor or nociceptin/orphanin FQ (N/OFQ) opioid peptide receptor (NOPr) was found in several rat brain areas, including the cerebral cortex, thalamus, subforn ical organ, habenula, hypothalamus, central gray, dorsal raphe, locus coeruleus hippocampus, amygdala, caudate nucleus, putamen, medial thalamic nuclei, and the dorsal horn of the spinal cord.^14^, ^17^

Most of opioid peptides do not penetrate the blood–brain barrier (BBB) so their effects, when administered intravenously, are associated with activation of peripheral ORs.^22^, ^23^, ^24^ However, the nonpeptide OR agonists can enter the brain and activate ORs in autonomic centers regulating the functional state of the heart. It has been shown that perfusion of the fourth cerebral ventricle with the selective peptide μ OR agonist FK 33–824 induces bradycardia in the conscious dogs.^25^ In anesthetized dogs, [D‐Met^2^,Pro^5^]enkephalinamide perfusion through the cerebroventricular system elicited bradycardia, which was accompanied by an increase in the vagal discharge rate.^26^ It has been shown that intracisternal administration of opioid peptides also evoked bradycardia in unanaesthetized dogs.^27^ This effect was abolished by pretreatment with atropine. It has also been found that intracerebroventricular administration of the selective μ OR agonist DAMGO or the selective δ OR agonist DPDPE increased plasma catecholamine levels and blood pressure (BP) in conscious rats.^28^ However, DAMGO appeared to be a more potent regulator of the catecholamine level than DPDPE. At a dose of 5 nM and higher, DAMGO induced bradycardia mediated by vagal activation. The authors concluded that brain ORs regulating autonomic outflow, cardiovascular and respiratory function are mainly of the μ type, although a δ opioid system may also contribute to sympathoadrenal and respiratory effects of opioids. Thus, presented data indicate that ORs are present in the brain regions responsible for the regulation of function of the cardiovascular system and the stress response to strong stimuli.

### B. Opioid Receptors in the Heart

All three OR (μ, δ, κ) transcripts were also detected in several peripheral tissues including the intestine, adrenal, kidney, and lung.^29^ In the stomach, δ OR and κ OR but not μ OR transcripts were found.^29^ mRNAs for opioid precursors were detected in adrenocortical cells.^30^ It has been established that μ and κ OR agonists can regulate cortisol and aldosterone secretion from the adrenocortical cells.^30^ The δ OR was found in a PC12 cell line derived from a pheochromocytoma of the rat adrenal medulla.^31^ Changes in function of these organs by activation of their ORs may indirectly affect the heart's function.

The first article reporting the existence of ORs in the myocardium was published in 1981.^32^ In 1988, the existence of δ OR in the myocardium was demonstrated using a radioligand binding assay.^33^ The next year, δ and κ ORs were also found in rat cardiac sarcolemma using this method.^34^ Later other investigators^35^ confirmed the existence of κ_1_ OR in the myocardium.^36^ Opioid‐binding sites in the myocardium were also confirmed in other studies.^37^, ^38^ In 1996, transcripts of δ and κ ORs were found in the heart.^29^ These data were later confirmed by Weil et al.^39^ None of these studies detected the μ OR in cardiomyocytes. However, in 1995, the μ_3_ subtype of this receptor was detected in the coronary microvascular's endothelial cells.^40^ This group of researchers also established that endothelial cells express a δ_2_ OR subtype.^41^ Vascular smooth muscle cells also appeared to express δ OR.^42^ Dumont and Lemaire were able to detect and characterize a high‐affinity [^3^H]nociceptin binding site in the membrane preparations of rat heart.^43^ Kim et al. confirmed the existence of the ORL1 receptor in cardiac myocytes.^44^ Thus, the view was formed that cardiac myocytes express δ OR, κ OR, and ORL1 receptor but not μ OR. This opinion was changed in 2005 when Head et al. found μ OR on the sarcolemma of cardiomyocytes using immunofluorescence microscopy.^45^ μ‐OR mRNA was also identified in the human right atrium. However, the amount of this receptor's mRNA in cardiomyocytes was significantly lower than the ORL1 mRNA content.^46^ Later, μ, δ, and κ ORs were detected immunohistochemically in human heart.^47^ The researchers found that μ and δ ORs are located mainly in cardiomyocytes as well as on sparse individual nerve fibers. Likewise, κ OR was identified predominantly in cardiomyocytes. This receptor was also found on intrinsic cardiac adrenergic (ICA) cells. It has been established that the δ OR is colocalized with the sensory neuron marker calcitonin gene‐related peptide (CGRP).^47^ Previously, similar data were obtained by Mousa et al. They identified μ OR and κ OR mRNA, as well as other OR proteins on cardiac parasympathetic, sympathetic, and sensory neurons.^48^ δ ORs were detected in the cholinergic neurons, small intensely fluorescent catecholaminergic cells, afferent nerve terminals, and atrial cardiomyocytes.^49^

Thus, all four types of ORs (μ, δ, κ, and ORL1) have been found in cardiomyocytes. δ OR, κ OR, and ORL1 receptor appeared to have the highest density in cardiomyocytes. μ and δ ORs are present in the endothelial cells and vascular smooth muscle cells express δ OR. ORs have been detected on the sensory nerve terminal, on ICA cells and are probably present in the sympathetic and parasympathetic terminals in the heart. It is safe to assume that activation of any of these receptors may potentially affect the functional state of the heart.

ICA cells were identified in rodent and human heart by Huang et al.^50^ In 2007, they discovered localization of δ OR immunoreactivity in ICA cells in human and rat hearts.^51^ They demonstrated that the selective δ_1_ OR agonist DPDPE enhanced epinephrine^51^ and CGRP release^52^ from ICA cells in denervated rat heart and these effects were abolished by the β‐adrenergic and CGRP receptor inhibitors. The authors suggested that the cardiotropic effects of δ OR agonists are mediated through β_2_‐AR/CGRP signaling.

### C. Opioid Receptors Modulate Neural Control of the Heart

Opioid peptides can alter the autonomic nervous regulation of the heart function. Indeed, Kett et al. established that intravenous administration of H‐Tyr‐D‐Arg‐Phe‐Lys‐NH_2_ (DALDA), a selective peptide μ OR agonist that does not penetrate the BBB, blunted norepinephrine‐induced baroreflex bradycardia but had no effect on the sodium nitroprusside‐evoked tachycardia.^53^ Pretreatment with naloxone methiodide, a peripheral OR antagonist, abolished DALDA‐induced suppression of baroreflex. These data indicate that DALDA inhibits the baroreflex through peripheral OR occupancy. Later, these investigators established that the selective peptide μ OR agonist D‐Ala^2^,N‐Me‐Phe^4^,Gly^5^‐ol (DAMGO) suppresses baroreflex‐mediated bradycardia in the awake sheep but the selective κ OR agonist U50,488 had no such effect.^54^ Peripheral μ OR stimulation can suppress vagus‐mediated baroreflex and it can be assumed that these ORs are located in the nerve endings innervating the sinoatrial node.

Urthaler et al. established that selective perfusion of the sinus node with morphine in anesthetized dogs evokes bradycardia.^55^ Bradycardia was not altered by atropine or vagotomy and intranodal administration of morphine had no effect on the acceleration of heart rhythm produced by stellate ganglion stimulation or by selective perfusion of the sinus node with norepinephrine. The authors concluded that morphine‐evoked bradycardia was autonomic nervous system independent and a direct effect of morphine on the sinoatrial node cells. These results were confirmed by the data of Gautret and Schmitt.^56^ They found that an intravenous administration of ethylketocyclazocine, a preferential κ OR agonist, induced a fall in heart rate (HR) and BP in rats anaesthetized with pentobarbital. The bradycardia and the hypotension were not altered by bilateral vagotomy and atropine, but were completely eliminated by naloxone and Mr 2266, a preferential κ OR antagonist. Ethylketocyclazocine‐induced bradycardia persisted in β‐adrenoreceptor‐blocked and pithed rats.^56^ These results indicate that peripheral κ OR located in the heart's conduction system can affect cardiac rhythm.

However, other data indicate that opioids can exhibit vagolytic effect. The *nervi vagi* of isolated perfused rabbit heart were electrically stimulated and morphine, a preferential μ OR agonist, met‐enkephalin, μ OR and δ OR agonist, and D‐Ala^2^,D‐Leu^5^‐enkephalin (DADLE), a preferential δ OR agonist, reduced the vagal bradycardia with IC_50_ values of 148, 25, and 3.2 nM, respectively. Pretreatment with naloxone abolished the vagolytic effect of all opioids. The selective δ OR antagonist ICI 174864 eliminated met‐enkephalin effect but did not antagonize morphine's action.^57^ These data indicate that stimulation of both μ OR and δ OR can attenuate vagus‐mediated bradycardia but stimulation of presynaptic δ OR have a more powerful vagolytic effect. Similar data were obtained by Musha et al. in the experiments on anesthetized dogs with electrical stimulation of *n. vagus*.^58^ They confirmed that presynaptic δ OR activation prevents vagal bradycardia. In pithed rats pretreated with propranolol, vagal stimulation or injection of methacholine decreased HR.^59^ The selective ORL1 receptor agonist nociceptin (orphanin FQ) decreased the vagal bradycardia but did not modify the methacholine‐induced decrease in HR. The selective ORL1 receptor antagonist [Phe^1^ψ(CH_2_‐NH)Gly^2^]‐nociceptin(1‐13)NH_2_ antagonized vagolytic effect of nociceptin. Authors concluded that orphanin FQ prevents vagal bradycardia acting on the presynaptic ORL1 receptor located on the vagal terminal in the heart.^59^ It was established that MEAP (met‐enkephalin‐Arg‐Phe) and the selective δ_2_ OR agonist deltorphin II suppressed vagal bradycardia when they were delivered directly into the sinoatrial node by local microdialysis.^60^ The authors also found that δ OR stimulation only in the sinoatrial node prevents vagal bradycardia. In the further study, they conducted a comparative analysis of the ability of δ OR agonists to suppress vagal bradycardia during administration into the sinoatrial node and found that the vagolytic effect of opioids is mediated by δ_2_ ORs in the sinoatrial node.^61^ These data were confirmed in a subsequent study by the same group.^62^ They later established that δ_2_ ORs are located on the cholinergic vagal terminals in the sinoatrial node.^63^

Opioids can modulate not only the vagal discharge rate but also sympathetic outflow. Ledda and Mantelli using isolated guinea‐pig atria discovered that the nonselective OR agonist etorphine inhibits the sympathetic response induced by direct electical stimulation.^64^ Pretreatment with naloxone abolished this effect of etorphine. However, etorphine did not affect an inotropic effect of norepinephrine. Authors concluded that etorphine stimulates presynaptic inhibitory ORs on adrenergic nerve terminals in the heart. Later they established that the inhibitory effect of the opioid peptides could be due to stimulation of presynaptic inhibitory δ and κ ORs on adrenergic nerve terminals in the heart.^65^, ^66^ Somewhat different results were obtained by Starke et al.^67^ In their study using selective OR agonists and antagonists they found that, under in vitro conditions, only presynaptic κ ORs but not μ ORs or δ ORs inhibit the norepinephrine release from the sympathetic nervous innervating the rabbit heart. Fuder published results of experiments where isolated guinea‐pig atria were loaded with ^3^H‐(–)‐norepinephrine. In these experiments, the intrinsic nerves stimulation evoked norepinephrine efflux.^68^ They discovered that the nonselective OR agonist etorphine, the κ OR agonists ethylketocyclazocine, dynorphin A (1‐13), and the δ OR agonist DADLE but not the preferential μ OR agonist morphine inhibit the stimulation‐induced norepinephrine efflux in a concentration‐dependent manner. The inhibitory effect of ethylketocyclazocine and etorphine was antagonized by naloxone. The authors hypothesized that activation of presynaptic κ ORs and apparently δ ORs inhibits norepinephrine release from sympathetic nerves in the heart.^68^ Others showed that a strong inhibition of on the sympathetic‐mediated positive inotropic effect evoked by electrical field stimulation of guinea‐pig atria can be achieved by κ OR agonists U‐50488 and U‐69593, whereas δ OR agonists, DPDPE and BW373U86, were ineffective. This effect of κ OR agonists was reversed by the selective κ OR antagonist nor‐binaltorphimine.^69^

Similar data were obtained in the in vivo experiments on pithed animals. Thus in pithed rabbits, it was found that ethylketocyclazocine decreased BP, the endogenous plasma norepinephrine level, and the 3H‐norepinephrine release rate.^70^ These effects were inhibited by naloxone. Investigators concluded that ethylketocyclazocine inhibits norepinephrine release from postganglionic sympathetic neurons, apparently by stimulation of ORs at the terminal axons. Later they established that the preferential κ_2_ OR agonist bremazocine prevents the 3H‐norepinephrine release and BP elevation in response to electrically (2 Hz) stimulated sympathetic outflow in pithed rabbits but has no effect on the BP increase evoked by an intravenous infusion of norepinephrine.^71^ The inhibitory effects of bremazocine were antagonized by naloxone. These results indicate that the κ_2_ OR stimulation inhibits norepinephrine release and consequently lowers BP by activation of peripheral, probably prejunctional, κ OR. This function of κ OR was later confirmed in the experiments of Caffrey's group,^72^ while Feuerstein et al. excluded a possible role of μ OR in the regulation of sympathetic outflow in pithed rats.^73^ Malinowska et al. showed in the experiments on pithed rats pretreated with atropine that the postganglionic sympathetic nerves innervating the rat heart have presynaptic ORL1 receptor and its activation inhibits the sympathetic outflow.^59^ Thus, the data show that activation of peripheral ORs can inhibit the cardiotropic effects of parasympathetic and sympathetic nerve stimulation. However, in the conscious animals the effect of opioid peptides can be quite the opposite. In particular, it has been shown that in unanesthetized sheep and dogs, intravenous administration of enkephalins or nociceptin may cause transient rise in BP and HR associated with enhanced sympathetic outflow.^74^, ^75^, ^76^ These effects were associated with activation of ORs located outside the BBB in the area postrema, a BBB‐deficient small, elevated area in the lateral wall of the inferior recess of the fourth ventricle.^74^, ^75^, ^76^ It was established that stimulation of central μ and δ OR also can increase plasma catecholamine levels and BP.^12^, ^28^

In 1990, Giuliani et al. demonstrated that electrical stimulation of the left atria of reserpine‐pretreated guinea pigs in the presence of atropine produces a positive inotropic effect involving activation of capsaicin‐sensitive afferents.^77^ μ OR agonists dermorphin, DAMGO, and morphine all inhibited this effect. The authors concluded that capsaicin‐sensitive nerves in the atrium have μ OR, which inhibit transmitter release from sensory nerve terminals.^77^ In a similar model, these investigators found that the selective ORL1 agonist nociceptin inhibits a positive inotropic response induced by electrical field stimulation.^78^ However, nociceptin (the selective ORL1 agonist) did not affect the positive inotropic effect of exogenous CGRP. Therefore, the authors suggested that nociceptin inhibits CGRP release by activation of ORL1 receptors localized on the afferent nerve endings in atria.^78^

The adrenal medulla can also be involved in the cardiovascular effects of opioids. Gulati and Bhargava studied cardiovascular effects of intravenous administration of κ OR agonists bremazocine, tifluadom, and U‐50,488 in anesthetized rats.^79^ All three opioids evoked bradycardia. Bremazocine and U‐50,488 decreased BP. The hemodynamic effects of the opioids were blocked by bilateral adrenal demedullation. The peripherally acting OR antagonist naltrexone methylbromide blocked the cardiovascular effects of U‐50,488. Based on these results, the investigators suggest that cardiovascular effects of κ OR agonists are mediated through the adrenal medulla and peripheral κ OR stimulation.^79^ The mechanism of this effect of κ OR agonists remains unknown.

Taken together, the available experimental data suggest that the heart is richly populated with ORs located on the sarcolemma of cardiomyocytes, cell membrane of ICA, and the coronary endothelial cells. In addition they are located on the sympathetic and parasympathetic nerve terminals in the heart, in the adrenal medula, and in the brain regions responsible for the regulation of the heart. Thus, it should come as no surprise that some of these can exert a cardioprotective effect against I/R injury.

## 3. ANTI‐INFARCT EFFECT OF PRETREATMENT WITH OPIOID RECEPTOR AGONISTS

### A. δ_1_ Opioid Agonists

Rats given 0.3 mg/kg of morphine intravenously prior to coronary artery occlusion/reperfusion experienced a decrease in the IS/AAR by 4.5‐fold.^10^ A year later, the same group of researchers found that the infarct‐sparing effect of morphine depended upon δ OR activation.^80^ In 1998, Miki et al. reported that morphine reduced infarct size in rabbits.^81^ Morphine was tested at doses of 0.3, 0.8, and 3 mg/kg but only the highest dose protected suggesting a species difference between ORs in rats and rabbits. Morphine also increases the tolerance of isolated rat cardiomyocytes to a 90‐min hypoxia.^82^ Wu et al.^83^ confirmed the cardioprotective properties of morphine. They administered 8 mg/kg intraperitoneally to rats. It was not mentioned why they selected such a high dosage but they found that morphine's protection could be prevented by blocking μ, δ, or κ ORs suggesting that all three OR subtypes seem to be involved in the cardioprotection of morphine. Lu et al. corroborated the infarct‐sparing effect of morphine in rats at 0.3 mg/kg intravenously.^84^

Bilir et al. showed that tramadol, an agonist and antagonist of ORs, increases the isolated rat heart's tolerance to I/R.^85^ In a clinical trial tramadol was given prior to coronary artery bypass surgery.^86^ Surprisingly, tramadol caused an increase in a marker of cardiomyocyte necrosis, cardiac troponin I (cTnI) in the blood of patients suggesting that this opioid actually exacerbates injury of the heart during coronary artery bypass surgery. This demonstrates why results of any animal study must be tested in clinical trials.

Irwine's group was the first to demonstrate remifentanil‐induced cardioprotection in both in vivo and isolated heart models.^87^, ^88^ The infarct‐reducing effect of remifentanil was abolished by pretreatment with the selective κ OR antagonist nor‐binaltorphimine and the selective δ OR antagonist naltrindole. Later, the cardioprotective effect of remifentanil was confirmed in an isolated perfused rat heart.^89^ In 2010, a clinical trial of remifentanil was carried out.^90^ Forty patients with on‐pump coronary artery bypass surgery were included in this trial. All patients were anesthetized with propofol and pretreated with fentanyl. Some of the patients (*n* = 20) received remifentanil (1 μg/kg intravenously and then infusion with rate of 0.5 μg/kg during 30 min) prior to surgery. Cardioprotection was determined 24 h postoperatively by assessing biochemical markers of myocardial necrosis: creatine kinase MB (CK‐MB) and cTnI. CK‐MB and cTnI levels were significantly lower in patients that received remifentanil.^90^ Thus, unlike tramadol, remifentanil appears to be cardioprotective not only in animals but also in patients with I/R injury of heart.

Pretreatment with the selective δ_1_ OR agonist TAN‐67 (10 mg/kg intravenously) decreased the IS/AAR in rats and the selective δ1 OR antagonist BNTX abolished the effect.^91^ This experiment indicated that the δ1 OR was protective and a year later, using isolated perfused hearts, it was shown that the δ OR‐selective agonist DADLE could also protect.^92^ More recent studies indicate that 10 mg/kg DADLE prior to coronary artery occlusion decreases IS/AAR and the highly selective δ OR antagonist naltrindole abolished this effect.^93^ The cardioprotective effect of DADLE was confirmed in later investigations.^94^, ^95^ In vivo, this peptide exhibited an infarct‐reducing effect in rats at a dose of 1 mg/kg.^94^ It was also found that the μ OR‐selective agonist methadone (0.3 mg/kg) shows an infarct‐reducing effect, which is actually mediated via δ OR activation.^96^

Takasaki et al. found that cardiomyocytes tolerance to hypoxia/reoxygenation is increased after addition the μ and δ OR agonist met‐enkephalin to the incubation buffer.^97^ Later, this team of investigators using naltrindole showed that the cytoprotective effect of met‐enkephalin is mediated via δ OR occupancy.^98^ Infusion of met‐enkephalin to rabbits starting 24 hr before coronary artery occlusion with an osmotic minipump promoted a decrease in the IS/AAR by 60%.^99^ However, a 24‐hr infusion of met‐enkephalin in mice failed to reduce the infarct size.^100^ This indicates again that there are species differences in the response to some opioids. This is most likely due to small but important differences in the genetic codes for these receptors among the species. In in vivo experiments with pigs, researchers could not demonstrate an infarct‐reducing effect of DADLE at a dose of 1 mg/kg intravenously.^101^

The ability of the δ_1_ OR agonist TAN‐67 to mimic the cardioprotective effect of preconditioning in rat heart was confirmed in the later studies both in vivo^102^ and in vitro.^103^, ^104^, ^105^ We established that perfusion of the isolated rat heart with the δ_1_ OR‐selective agonist DPDPE (154 nM) decreases reperfusion‐induced creatine kinase release.^106^ Pretreatment with the δ OR‐selective antagonist naltrindole (1 nM) completely abolished DPDPE's cardioprotective effect. In 2001, McPherson and Yao^107^ showed that the δ‐selective agonist BW373U86 (10 pM) increases tolerance of isolated cardiomyocytes to hypoxia/reoxygenation. The cardioprotective property of TAN‐67 and BW373U86 were confirmed in vivo at coronary artery occlusion and reperfusion.^108^, ^109^ In addition, it was established that the infarct‐sparing effect of BW373U86 (1 mg/kg) is a consequence of δ_1_ OR activation.^109^

In pigs, an infarct‐reducing effect of DPDPE was found at a dose of 1 mg/kg intravenously^101^ but this dose was not protective in rats.^110^, ^111^ Again, a species difference was present. DPDPE at the final concentration of 0.1 mg/L (154 nM) did protect the isolated perfused rat heart^112^, ^113^ and DPDPE's protection in rat heart can be blocked by naltrindole.^104^, ^106^, ^114^ The infarct‐sparing effect of DPDPE was confirmed in experiments in the isolated rat heart by Huang et al.^52^ In 2006, Watson et al. reported that the δ_1_ and δ_2_ OR agonist ARD‐353 (0.3 mg/kg) decreased the IS/AAR in rats.^115^ This effect disappeared after δ_1_ OR inhibition with BNTX. In addition, Watson et al. obtained data that ARD‐353 does not penetrate through the BBB.^115^ These authors concluded that the cardioprotective effect of ARD‐353 is a consequence of peripheral δ_1_ OR activation. In 2006, Gross et al.^116^ reported that the δ OR agonist fentanyl isothiocynate caused infarct reduction at a dose of 10 μg/kg intravenously. Hence, there is a reason to believe that the δ_1_ OR agonists are excellent candidates for cardioprotective drug development.

### B. δ_2_ Opioid Agonists

In 2002, in experiments with pigs, we demonstrated the infarct‐reducing effect of the putative δ_2_ OR agonist deltorphin D at a dose of 1 mg/kg.^101^ In 2009, in experiments with rats, we confirmed these data.^117^ The selective δ_2_ OR agonist deltorphin II at a dose of 0.12 mg/kg can decrease the IS/AAR. The infarct‐sparing effect of deltorphin II was maintained in the presence of the δ_1_ OR antagonist BNTX but disappeared after δ_2_ OR block with naltriben.^117^ The cardioprotective effect of deltorphin II was abolished after blocking peripheral OR with naloxone methiodide (5 mg/kg). Hence, the δ_2_ OR also seemed to be protective.

### C. κ Opioid Agonists

The κ_1_ OR‐selective agonist U50,488 increases the tolerance of isolated cardiomyocytes to sodium cyanide toxicity in the incubation buffer.^118^ They showed that U50,488 (10 μM) decreases the IS/AAR in the isolated perfused rat heart. We confirmed that pretreatment with U50,488 protects the isolated perfused rat heart against global I/R.^113^, ^119^, ^120^ Addition of κ OR‐selective agonist dynorphin to incubation buffer increases tolerance of rabbit cardiomyocytes to 3 hr of hypoxia and the κ OR‐selective antagonist GNTI abolished dynorphin's protection.^98^ In 2004, it was shown that pretreatment with the κ OR agonists U50,488; ICI 204,448; and BRL 52537 exhibit infarct‐reducing effect in vivo.^121^ The κ OR‐selective antagonist nor‐binaltorphimine abolished the infarct‐sparing effect of U50,488 and ICI 204,448 but did not affect the cardioprotective effect of BRL 52537.^121^ Since ICI 204,448 does not penetrate through the BBB,^122^ it is likely that the peripheral κ OR activation promotes the protection. The antinecrotic effect of U50,488 is seen in the isolated heart. Recently, we have found that the quaternary ammonium salt of U50,488 (Q‐U50,488), which is not able to pass the BBB, elicits a protective effect against cardiac I/R injury.^123^ The infarct‐sparing effect of Q‐U50,488 was abolished by nor‐binaltorphimine implicating a peripheral κ OR. It can safely be assumed that the κ OR regulating cardiac tolerance to I/R is located in the heart.

### D. μ Opioid Agonists

Using isolated perfused rat heart we found that the μ OR‐selective agonist DAMGO reduces infarction after global I/R.^124^ The protection from DAMGO was confirmed in our later investigations.^125^, ^126^ In addition, we established that the μ OR‐selective agonist DALDA also can prevent cardiac cell death during global I/R. However, intravenous DAMGO (0.1 mg/kg) or DALDA (0.1 mg/kg) 15 min prior to heart isolation actually increased the injury from I/R ex vivo.^125^, ^126^, ^127^ Gross's group found that DAMGO (0.1 mg/kg intravenously) had no effect on the IS/AAR in rats after I/R in vivo.^128^ In our in vivo investigation in rats with coronary artery occlusion (45 min) and reperfusion (2 hr), we studied the μ OR‐selective agonist dermorphin H (0.12 mg/kg) and DAMGO (0.08 or 0.8 mg/kg).^110^, ^111^ Neither of these peptides had any effect on the IS/AAR. It remains unclear why DAMGO is so protective ex vivo but not in vivo.

Gross et al. found that the μ and δ OR‐selective agonist Eribis peptide 94 starting 10 min after coronary artery occlusion (30 min) and continuing during reperfusion decreases the IS/AAR in open‐chest rats. This protection persisted after inhibition of δ OR with naltrindole, δ_1_ OR with BNTX, and κ OR with nor‐binaltorphimine. However, it was abolished with the selective μ OR antagonist CTOP.^129^ This was the first convincing evidence that μ OR activation protects the heart from I/R.

### E. ORL1 Opioid Agonists

Recently, we evaluated a fourth OR subtype that usually denoted as ORL1 receptor (opioid‐like receptor 1) in an in vivo rat model using the endogenous ORL1‐selective agonist nociceptin. Neither 0.4 or 2.2 mg/kg had any effect on the IS/AAR.^110^, ^111^ In our opinion, it is too early to draw a final conclusion that ORL1 receptors do not affect the heart's tolerance to I/R because we have not yet studied it in the isolated heart. The μ OR agonists are cardioprotective ex vivo but not in vivo and it is possible that nociceptin may act in a similar fashion.

## 4. TOXICITY OF HIGH‐DOSE OPIOIDS AND THE CARDIOPROTECTIVE EFFECTS OF OR ANTAGONISTS

The aforementioned studies demonstrate that OR activation can increase the heart's tolerance to I/R but there are some studies demonstrating that OR stimulation can also exacerbate I/R injury. Intravenous administration of morphine at a dose of 2.1 mg/kg can induce ST segment depression in patients with ischemic heart disease.^130^ The authors interpreted this effect as a manifestation of myocardial ischemia. In another study, it was shown that morphine at a dose of 1 mg/kg increases ST segment elevation in cats with coronary artery ligation, which they regarded as worsening of the heart's ischemia.^131^ In 1982, the same group of authors obtained data that morphine can increase the infarct size in rats.^132^ Morphine was administered at a dose of 3 mg/kg intravenously for 10 min prior to a 48‐h coronary artery occlusion without reperfusion. Permanent occlusion without reperfusion in rodent hearts is now considered as an invalid methodology for evaluating cardioprotection since cardiac muscle cannot survive in the complete absence of blood flow. In addition, these data contradict the results of the Chinese investigators, which showed that morphine at a dose of 8 mg/kg evokes a decrease in the IS/AAR.^83^ We tested 0.3, 0.8, and 3.0 mg/kg morphine pretreatment in open‐chest rabbits and found no effect of the two lower doses but greatly reduced infarct size with the high dose.^81^

In 1985, it was reported that 1.1 or 3.6 mM naloxone in the perfusion solution protects the isolated heart.^133^ Naloxone's IC_50_ toward μ and δ OR is 8.2 nmol.^134^ Similarly, the *K*_i_ of naloxone toward μ OR is reported to be 3.4 nmol but that toward δ OR is 50 nmol.^135^ We would suggest that the cardioprotective effect of their very high‐dose naloxone is probably a nonspecific membrane stabilizing effect of the drug, rather than a consequence of the blockade of ORs. It should also be noted that in most of our experiments we have not observed infarct‐reducing effect of naloxone, naltrexone, and most of other OR antagonists.^81^, ^110^ Similarly, many other investigators failed to observe a cardioprotective effect of the OR antagonists in situ or in vitro. An exception is the work of Chen et al. They performed 45‐min global ischemia and 60‐min reperfusion of an isolated rat heart in which OR antagonists were added to perfusion buffer for the first 10 min of reperfusion. Necrosis was evaluated by IS/AAR and by monitoring CK‐MB levels in coronary effluent. Naloxone (10 nM), naltrindole (5 nM), or nor‐binaltorphimine (5 nM) decreased the IS/AAR and CK‐MB release.^136^ Hence, these OR antagonists mimic IPost phenomenon. The concentrations of antagonists indicators used approach their published *K*_i_ and IC_50_.^134^, ^135^, ^137^ Therefore, we cannot easily dismiss the cardioprotective effect of OR antagonists as a nonspecific effect. We found that intravenous administration of the μ OR antagonist CTAP (1 mg/kg) to rats prior to coronary artery occlusion (20 min) and reperfusion (3 hr) promotes a decrease in the IS/AAR.^138^ However, protection may have been mediated via the somatostatin receptor for which this peptide exhibits moderate affinity.^139^ Somatostatin is known to limit the IS/AAR in in vivo studies.^140^

Our investigations do indicate the existence of an OR pool, or non‐ORs, whose activation with opioids negatively affects cardiac tolerance to I/R.^104^, ^112^ In isolated rat heart studies, we observed that the cardioprotective effect of the δ_1_ agonist DPDPE disappears if the concentration of peptide in the perfusion buffer is increased to 740 nM.^104^, ^112^ In isolated murine heart, 10 μM of morphine was not protective^141^ while 0.3 μM did protect isolated rabbit heart.^81^ It seems highly likely that concentration of 10 μM was so high that morphine began binding to a pool of receptors that negatively affected the heart's tolerance to I/R. Gross's group was unable to protect hearts with 1 μM BW373U86 in isolated murine hearts^141^ while BW373U86 did protect isolated chick cardiomyocytes but only at a concentration of 10 pM.^107^ Mixing species always complicates interpretation but an obvious explanation is that overdosing can lead to negative off target effects. We recommend that ex vivo and in vitro experiments should test agonists at a concentration tenfold higher than the *K*_i_ or EC_50_.^81^ They should also be aware that the binding affinities of these drugs can vary widely among species.

Aitchison et al. reported that DADLE at 10 nM exhibits infarct‐sparing that was diminished at 1 μM. Inhibition of κ OR with nor‐binaltorphimine restored the full cardioprotective effect of high concentration DADLE.^142^ The authors concluded that the diminished effect of DADLE at high concentration is due to activation of κ OR. These data closely resemble our results with DPDPE.^104^, ^112^ In addition, Aitchison et al. established that the nonselective κ OR agonist bremazocine (30 nM) ex vivo increased infarct size.^142^ This negative effect of bremazocine disappeared after inhibition of κ OR. In this regard it should be noted that U50,488 is the selective κ_1_ OR agonist but bremazocine is an agonist of κ_2_ OR.^143^ It seems reasonable to assume that the activation of κ_2_ OR exacerbates injury from I/R.

In 2005, Meine et al. published the results of a prospective, nonrandomized study, which included patients with acute coronary syndrome (ACS) with non‐ST‐segment elevation (NSTE; *n* = 57,039).^144^ The authors evaluated the outcome of patients treated with morphine and those who were not. It was found that treatment with morphine was associated with increased risk of in‐hospital mortality. The authors raised concerns about the safety of using morphine in patients with ACS NSTE but pointed out that that could only be answered with a randomized trial.^144^ These data were in accord with a few other studies.^130^, ^145^, ^146^ In particular, Conahan et al. demonstrated that morphine (2 mg/kg) caused severe hypertension and an increase in systemic vascular resistance in patients undergoing heart valve replacement.^146^ Later Lappas et al. reported that the addition of 5% NO to morphine (2 mg/kg intravenously) decreased BP, cardiac index, stroke index and increased pulmonary capillary wedge pressure.^146^ Intravenous administration of morphine at a dose of 2.1 mg/kg can induce ST segment depression in patients with ischemic heart disease.^130^ However, we would like to draw readers’ attention to the fact that an extremely large dose of morphine (2 mg/kg) was used in these three studies.^130^, ^145^, ^146^ Indeed, in current cardiological guidelines, the recommended dose of morphine is 4–8 mg (0.05–0.1 mg/kg).^147^ Experimental studies suggest that morphine has the infarct‐limiting effect at a dose of 0.3 mg/kg.^10^ It comes as no surprise that morphine can provide an adverse effect on the cardiovascular system at a dose many times exceeding the therapeutic dose. In cardiological practice, morphine and fentanyl are used not only for pain relief in patients with AMI but also to ease anxiety, reduce preload, due to venodilation,^147^, ^148^, ^149^ and afterload, due to reducing systemic vascular resistance.^148^, ^150^ It has been established that morphine and fentanyl can decrease myocardial oxygen consumption and reduce lactate production by the left ventricle in human.^151^ Both of those effects may increase cardiac resistance to ischemia and improve the outcome in AMI. Morphine is also used for the prevention of pulmonary edema, and cardiogenic shock.^152^ Therefore, morphine and other opioids are prescribed in the most serious cases characterized by a higher mortality than in patients with mild AMI, like those included in the study of Meine et al.^144^ An overdose of opioids may cause a depression of respiration, hypotension, and vomiting.^152^

In summary, pretreatment with agonists of μ, δ_1_, δ_2_, and κ_1_ OR exhibit cardioprotective properties both in vivo and in vitro. These pharmacological agents mimic the preconditioning phenomenon. The role of the ORL1‐receptors in this regard remains open, however. A number of reports points to the existence of important species differences in the reaction of infarcted myocardium to opioids. Some receptors, such as κ_2_ ORs, may actually exacerbate the ischemic and reperfusion heart injury. But the agonists of μ, δ_1_, δ_2_, and κ_1_ ORs can be considered as the most promising group of agents able to induce cardioprotection. This opinion can be supported by numerous studies.^80^, ^87^, ^93^, ^96^, ^98^, ^106^, ^109^, ^110^, ^115^, ^116^, ^121^, ^128^, ^129^

## 5. ANTIAPOPTOTIC EFFECT OF THE OPIOID RECEPTOR AGONISTS

It is well known that reperfusion induces enhancement of production of reactive oxygen species (ROS)^153^ and Ca^2+^ overload in cardiomyocytes.^154^ Calcium ions and ROS evoke opening of MPT (mitochondria permeability transition pore), which is a protein supramolecular complex built into the inner mitochondrial membrane.^155^, ^156^ Opening of this pore collapses the potential across the inner mitochondrial membrane, which prevents ATP generation by the mitochondria. MPT also releases cytochrome c and AIF (apoptosis inducing factor) in the intermembrane space into the cytosol.^155^, ^156^ Cytochrome c together with APAF‐1 (apoptosis protease activating factor), procaspase‐9, and ATP form a supramolecular complex named apoptosome.^156^ The apoptosome catalyzes proteolysis of procaspase‐9 to become active caspase‐9, which in turn catalyzes the cleavage of other proteins ultimately leading to apoptosis, a process in which the cell is killed and digested from within over several days. Protein AIF activates translocation of endonuclease G from cytosol into nucleus where the latter catalyzes DNA fragmentation that is a characteristic of apoptotic cells.^156^ These events are developed mainly during the first minutes of reperfusion. Therefore, the opening of MPT is a major cause of death of cardiomyocytes after the restoration of coronary blood flow.^155^ Necrosis quickly ensues if too many mitochondria within the cell are lost to MPT and the cell becomes tetrazolium negative (popular marker for infarct size studies) minutes after reperfusion due to membrane failure. If only a small fraction of the mitochondria is involved, however, the cell may survive the initial I/R only to succumb to apoptosis a day or two later. Apoptotic cells are tetrazolium positive in the first hours of reperfusion. The evidence is strong that IP protects by inhibiting MPT formation at reperfusion.^157^ Generally, apoptosis and necrosis act in parallel and markers of apoptosis can be used to assess injury from I/R.

The first work indicating that opioids inhibit apoptosis of cardiomyocytes was published in 2001. Isolated chicken embryo cardiomyocytes were subjected to 12 h of hypoxia and 12 h of reoxygenation. Apoptosis was evaluated by the number of TUNEL‐positive cells (terminal deoxyribonucleotide transferase‐mediated dUTP nick end labeling). Fifty‐four percent of the cells were TUNEL‐positive. However, if BW373U86 (20 pM) was added to medium only 39% of the cells became apoptotic. The selective inhibition of δ_1_ OR with BNTX abolished the cytoprotective effect of BW373U86.^158^ Okubo et al. found that opioids can exert an anti‐apoptotic effect in vivo. Morphine at 0.3 mg/kg prior to coronary artery occlusion reduced the number of TUNEL‐positive cells in the heart from 12.4% in control to only 5.2%. The δ OR antagonist naltrindole (10 mg/kg intravenously) abolished the effect of morphine.^159^ These findings led the investigators to conclude that the antiapoptotic effect of morphine was dependent upon δ OR activation. The antiapoptotic effect of morphine on isolated cardiomyocytes was confirmed in later experiments.^160^ In isolated perfused rat hearts exposed to 30‐min global ischemia and 60‐min reperfusion 16% of the cells were TUNEL‐positive but 3 μM morphine in the perfusion solution decreased this index to 5%.^161^ In 2012, Kim et al. using isolated cardiomyocytes found that addition of remifentanil to the cell incubation medium prior to hypoxia/reoxygenation increased cell survival, decreased the concentration of Ca^2+^ in the cytoplasm, decreased activity of caspase‐3, increased anti‐apoptotic protein Bcl‐2 (B‐cell lymphoma protein‐2) over that in untreated cells.^162^ In 2009, it was noted that the selective κ_1_ OR agonist U50,488 causes an antiapoptotic effect.^163^ This study was performed in rats with coronary artery occlusion (45 min) and reperfusion (3 h). The κ_1_ OR agonist U50,488 was administered intravenously prior to ischemia. The number of TUNEL‐positive cells in the area of I/R was 21.3% but in animals receiving U50,488 this number dropped to12%. The selective κ OR antagonist nor‐binaltorphimine eliminated this effect indicating that the antiapoptotic effect of U50,488 was mediated via κ_1_ OR activation. We recently confirmed their hypothesis using Q‐U50,488, which does not crosses the BBB.^123^

The aforementioned studies suggest that δ and κ_1_ OR activation reduces the appearance of apoptosis of cardiomyocytes following reperfusion. It has not been determined whether agonists of μ OR and ORL1 receptors can prevent apoptosis of cardiomyocytes.

## 6. OPIOIDS CAN MIMIC DELAYED ISCHEMIC PRECONDITIONING

Fryer et al. found that 24 h after injection of TAN‐67, there was a return of protection against I/R. Combining TAN‐67 with the δ_1_ OR antagonist BNTX abolished this delayed protection. The authors concluded that the delayed protective effect of TAN‐67 is dependent upon δ_1_ OR activation.^164^ The delayed protective effect of TAN‐67 was confirmed in later works.^165^, ^166^ In 2004, it was found that the nonpeptide δ OR‐selective agonist SNC‐121 caused a delayed window of protection in rats and surprisingly its protection was retained after inhibition of ORs with naloxone.^167^ The authors concluded that the cardioprotective effect of SNC‐121 was not dependent on OR and illustrates the importance of testing with antagonists. Shinmura and colleagues found that the selective δ OR agonist BW‐373U86 can mimic delayed preconditioning.^168^ Other investigators found that the nonpeptide δ_1_ and δ_2_ OR agonist ARD‐353 (0.3 mg/kg) evoked delayed conditioning.^115^ Morphine (3 mg/kg) also triggered delayed conditioning^169^ as did morphine at a dose of 0.3 mg/kg.^170^ OR antagonists were not used in these two studies. Hence, the responsible for delayed protective effect OR was not identified.

A 30‐min incubation of isolated cardiomyocytes with U50,488 for 20 hr prior to hypoxia/reoxygenation increases cell tolerance to hypoxia/reoxygenation.^171^ This effect of U50,488 did not occur after κ OR inhibition with nor‐binaltorphimine. The delayed preconditioning phenomenon of U50,488 was confirmed in later works by the same authors.^172^, ^173^ Intravenous administration of remifentanil, a nonselective OR agonist, can induce a delayed cardioprotective effect^174^ and this was confirmed by other investigators.^175^ All three OR antagonists (CTOP, nor‐binaltorphimine, naltrindole) abolished infarct‐sparing effect of remifentanil.^174^ Participation of ORs in the delayed cardioprotective effect of remifentanil has been confirmed by Sun et al.^175^

## 7. INVOLVEMENT OF ENDOGENOUS OPIOIDS IN THE INFARCT‐REDUCING EFFECT OF REMOTE ISCHEMIC PRECONDITIONING

In 2001, Dickson et al. attempted to clarify the nature of the humoral factor(s) mediating the infarct‐reducing effect of remote ischemic preconditioning (RIPC).^176^ Preconditioning of isolated perfused rabbit hearts was reproduced by three 5‐min episodes of ischemia interspersed with 10 min of reperfusion. Coronary effluent was collected, purified, and concentrated using Sep‐Pak C‐18 columns. They demonstrated that concentrated coronary effluent introduced to other isolated rabbit hearts can protect these hearts against ischemia (40 min) and reperfusion (120 min). This protective effect was eliminated by pretreatment with naloxone.^176^ In the next study, isolated jejunal segments were subjected to 1 hr of simulated ischemia followed by 30 min of reoxygenation.^177^ Pretreatment with coronary effluent concentrate also improved contraction of the jejunal segments during reperfusion. Naloxone abolished the inotropic effect of the coronary effluent. Authors believe that coronary effluent contains opioids, which mediate a protective effect of RIPC.^177^ The authors hypothesized that the endogenous mediator of the cardioprotective action of RIPC is endogenous opioid peptide Met5‐enkephalin‐Arg6‐Phe7.^178^ Patel et al. hypothesized that mesenteric preconditioning evokes release of endogenous opioids that protect the heart against I/R.^179^ Rats were subjected to coronary artery occlusion (30 min) followed by reperfusion (2 hr). Experimental groups underwent occlusion of the mesenteric artery (15 min) followed by reperfusion (10 min). Pretreatment with naloxone abolished the protective effects of RIPC.^179^ These data indicate that mesenteric preconditioning evokes release of endogenous opioid peptides that protect the myocardium against I/R. Weinbrenner et al. assumed that infarct‐sparing effect mediated by infrarenal occlusion of the aorta (IOA) may be transmitted by endogenous opioids.^180^ They established that IAO protected against I/R and this was abolished by pretreatment with the selective δ1 OR antagonist BNTX (7‐benzylidenenaltrexone).^180^ These results indicate that the protection by RIPC is transmitted by δ1 OR occupancy. Another group induced RIPC in rats by three cycles of femoral artery occlusion (5 min) followed by reperfusion (5 min).^181^ They demonstrated that RIPC evokes increase in plasma dynorphin (a nonselective κ OR agonist), but not met‐enkephalin (a μ OR and δ OR agonist) level. Pretreatment with the selective κ OR antagonist nor‐binaltorphimine eliminated the infarct‐sparing effect of RIPC. The selective δ OR antagonist naltrindole had no effect on the remote preconditioning.^181^ Hence, endogenous κ OR agonists, apparently dynorphin, mediate the cardioprotective effect of RIPC. Later, Rehmi et al. reported the participation of endogenous opioids in RIPC.^182^ Rentoukas et al. showed that morphine in combination with RIPC reduced infarct size in patients with primary percutaneous coronary intervention while RIPC alone did not.^183^

Thus, today, there is no doubt that endogenous opioid peptides participate in the mechanism of the cardioprotective effect of RIPC. However, it remains unclear what kinds of ORs are involved in the RIPC phenomenon. The aforementioned Met5‐enkephalin‐Arg6‐Phe7 and dynorphin are unlikely mediators of RIPC since they are not resistant to enzymatic hydrolysis.^184^, ^185^

## 8. OPIOIDS MIMIC POSTCONDITIONING PHENOMENON

The aforementioned studies demonstrate the ability of opioids to protect when applied as a pretreatment. A major indication for cardioprotection is ACS where the patient presents with ischemia already in progress. That makes pretreatment impossible so a postconditioning drug intervention is needed. Because much of the cell death in the heart is from MPT that form at reperfusion, it is theoretically possible to protect against infarction right up to the time of reperfusion. IPost has been shown to limit infarct size.^7^ Is it possible that OR agonists at reperfusion might also protect?

There are a few publications indicating that the OR agonist can protect when administered at the end of the ischemic period. In one such study, rats were exposed to 1‐h coronary artery occlusion and 2‐h reperfusion. When 0.3 mg/kg morphine was administered intravenously 10 min prior to reperfusion it evoked a decrease in the IS/AAR from 45 to 30%.^186^ ARD‐353 administered after 30 min of ischemia at a dose of 0.3 mg/kg immediately before removing the ligature decrease the IS/AAR from 55 to 35%.^115^ Since ARD‐353 does not penetrate the BBB, these authors concluded that its infarct‐reducing effect is mediated via peripheral OR.^115^ Tsutsumi et al. studied mice with 30‐min coronary artery occlusion and 2‐h reperfusion. The δ OR agonist SNC‐121 (10 mg/kg) was administered intravenously 3 min before reperfusion. The control IS/AAR was 44% but only 24% in SNC‐12‐treated mice.^187^ This study did not evaluate the role of OR antagonists.

The OR agonists mimic IPost not only in vivo but also ex vivo. In one study, the isolated perfused rat heart was exposed to 45‐min global ischemia and 60‐min reperfusion.^136^ Morphine was added to the perfusion buffer at 0.3, 3, and 30 μM for the first 10 min of reperfusion. Necrosis was assessed by tetrazolium staining and by CK‐MB in coronary effluent. Morphine decreased the IS/AAR at 0.3 μM and more so at 30 μM. Pretreatment with naloxone or nor‐binaltorphimine attenuated the protection.^136^ Unfortunately, the OR antagonists (naloxone, naltrindole, and nor‐binaltorphimine) exerted a small but significant cardioprotective effect by themselves, which complicates the interpretation.

In 2008, Jang et al. studied isolated rat heart with 30‐min coronary artery branch occlusion and 2‐hr reperfusion. Either morphine (1 μM) or the δ OR agonist BW373U86 (1 μM) were added to the perfusion solution starting 5 min prior to reperfusion of the occluded coronary branch. The total duration of perfusion with agonists was 15 min. Both agonists decreased the IS/AAR by threefold.^188^ Pretreatment with naltrindole (100 μM) abolished the infarct‐sparing effect of both agonists. Unfortunately, the authors used naltrindole in a concentration sufficient to inhibit all OR subtypes.^135^ Using isolated perfused rat heart, Mourouzis et al. reported that 10 μM morphine can mimic IPost.^189^ The ability of morphine at 1 μM to postcondition was also reported elsewhere.^190^, ^191^ In vivo I/R experiments in rats showed that intravenous administration of U50,488 (0.1 mg/kg) 5 min prior to reperfusion promotes a decrease in the IS/AAR but U50,488 10 sec prior to reperfusion had no effect on the IS/AAR.^192^ They also studied U50,488 (100 nM) in an isolated murine heart. The κ OR agonist was added to Krebs‐Henseleit buffer at the beginning of reperfusion and it decreased the IS/AAR.^192^ They did not test OR antagonists. However, this does not invalidate their conclusion that U50,488 protected via κ_1_ OR because *K*_i_ of U50,488 for κ_1_ OR is 7.4 nmol but the *K*_i_ of U50,488 for μ OR is 256 nmol.^134^

Methadone administered to an in situ rat experiencing 30‐min ischemia at a dose of 0.3 mg/kg for 5 min prior to reperfusion reduced infarct size. But if the injection was performed 10 sec after removal of the ligature, no changes in the IS/AAR could be detected.^96^ If the duration of ischemia of the heart was 45 min, the injection of methadone 5 min before reperfusion also had no effect on the IS/AAR. The authors concluded that this opioid mimics IPost if it is administered 5 min before reperfusion.^96^ Remifentanil was infused intravenously for 5 min starting 5 min before reperfusion in rats with 30‐min coronary artery occlusion and 2‐hr reperfusion. The IS/AAR was reduced by a dose of 10 μg/kg. Blocking δ or κ OR but not the μ OR by the agonist CTOP eliminated the protection.^193^ The ability of remifentanil to simulate IPost phenomenon was confirmed in another study performed in the isolated perfused rat heart.^194^

In 2011, it was reported that 1 μg/kg of a tetrapeptide referred to by the authors as Eribis peptide 94 (EP94) decreased the IS/AAR in rats at reperfusion.^195^ These authors did not confirm a role of ORs in the infarct‐reducing effect of EP94 but the authors did note that EP94 is a μ and δ OR agonist. Such a high potency of EP94 is surprising. However, in a later study by the same authors, it was reported that EP94 had an infarct‐sparing effect at a dose of 25 μg/kg but had no effect on the infarct size at the dose of 1 μg/kg.^196^ A 2012 study indicated that sufentanil simulates the IPost phenomenon at a dose of 1 μg/kg.^197^ It is known that sufentanil is also a selective agonist of μ OR.^198^ A further increase in the dose of this opioid did not lead to an enhancement of the infarct‐reducing effect.^197^ These data were confirmed in a later paper by the same group.^199^ Unfortunately, these researchers did not test OR antagonists, therefore, it remains unclear whether the cardioprotective effect of sufentanil is depended upon μ OR activation. Most recently, in the experiments on isolated perfused rat heart, the nonselective OR agonist remifentanil at reperfusion was protective.^200^ The infarct‐reducing effect of this opioid was eliminated by naloxone but the investigators did not use any of the selective OR antagonists.

Thus, the aforementioned studies provide ample evidence that activation of δ and κ_1_ OR can postcondition the heart. It remains unclear whether agonists of μ OR and ORL1 are also protective at the time of reperfusion.

## 9. LOCALIZATION OF OPIOID RECEPTORS THAT PROTECT THE HEART FROM I/R

Studies on the isolated heart seem to indicate that the infarct‐limiting effect of opioids is associated with the occupancy of the cardiac ORs. However, one should pay attention to two facts: (i) most studies have used OR ligands that penetrate the BBB, and (ii) in some studies the OR agonists were used at very large doses.^81^, ^91^, ^187^ For example, TAN‐67 was used at a dose of 10 mg/kg.^91^ But according to Knapp et al. the *K*_i_ of TAN‐67 for δ OR is 0.65 nmol.^201^ For comparison, the *K*_i_ of morphine against δ OR is 49 nmol.^135^ One can assume that in order to limit the size of myocardial infarction, a larger dose of morphine would be required. However, it has been demonstrated that morphine is protective at a dose of only 0.3 mg/kg in rats.^9^ An interesting possible explanation of this paradox could be that TAN‐67 activates a central δ OR that remotely increases cardiac tolerance to I/R via neural pathways and the low penetration of the BBB for TAN‐67 requires a higher dose.

There is a direct evidence of participation of central ORs in cardioprotection. A rat study with 30‐min coronary artery occlusion and 90 min reperfusion showed that intrathecal administration of morphine (0.3 μg/kg) for 20 min prior to ischemia promotes a decrease in the IS/AAR.^202^ In a similar study in 2009, intrathecal pretreatment with morphine again protected rat hearts in a dose‐dependent manner.^203^ Intrathecal administration of CTOP, naltrindole, or nor‐binaltorphimine abolished the infarct‐sparing effect and the authors concluded that all three (μ, δ, and κ) ORs are involved in the cardioprotective effect of morphine.^203^ The infarct‐reducing effect of morphine during intrathecal administration was confirmed in 2010.^204^ A cardioprotective effect of morphine was seen when it was infused for the 5 min prior to reperfusion and could be blocked by inhibition of μ, δ, or κ OR.^205^

In a study in rats with coronary artery occlusion/reperfusion, morphine was administered intravenously at a dose of 0.3 mg/kg.^84^ Naloxone methiodide, which does not cross the BBB, was administered prior to morphine injection intravenously or intrathecally at a dose of 20 mg/kg or 20 μg/kg.^84^, ^206^ Regardless of the route of administration, naloxone methiodide abolished the infarct‐sparing effect of morphine. The authors concluded that morphine protected through both central and peripheral ORs.

In 2012, it was shown that intrathecal administration of morphine to rats decreased the IS/AAR by twofold and the autonomic ganglion blocker hexamethonium completely abolished the protection.^207^ They concluded that the cardioprotective effect of morphine was mediated via central OR stimulation and signaling through the autonomic nervous system. In 2014, it was found that intrathecal administration of μ OR agonist fentanyl evokes a decrease in the IS/AAR.^208^ These data also indicate that the infarct‐reducing effect of opioids following intravenous administration may not only be a consequence of activation of peripheral but also of central ORs. The infarct‐limiting effect of opioid peptide EP94 occurred after blockade of peripheral OR with naloxone methiodide but disappeared after blocking peripheral and central ORs with naloxone.^129^ These authors concluded that the infarct‐reducing effect of EP94 is mediated via central OR activation. This result was surprising because opioid peptides usually penetrate the BBB poorly. For example, the opioid peptide dalargin exerts central effect only at a dose of 500 μg/kg.^209^ But Gross et al. used EP94 at a dose of 1 μg/kg.^129^

Thus, central OR stimulation clearly can increase cardiac tolerance to I/R. On the other hand, there is ample data with isolated hearts that cardiac OR can also protect the heart. It remains unclear, therefore, to what extent the infarct‐limiting effect of opioids during intravenous administration is mediated via central OR activation.

## 10. EFFECT OF OPIOIDS ON RECOVERY OF CARDIAC CONTRACTILITY DURING REPERFUSION

The above studies primarily concentrated on myocardial necrosis as the endpoint. Cardiac injury also manifests itself as a reduction in postreperfusion cardiac contractility. That reduction can be from loss of muscle to necrosis or it can be due to stunning, which is a transient loss of contractility following I/R. Preservation of mechanical function after I/R is paramount in the setting of cardiac surgery. Therefore, some studies used cardiac contractility as their endpoint rather than infarction.

### A. μ OR

We found that intravenous administration of the μ OR‐selective agonists DALDA (0.1 mg/kg) or DAMGO (1 mg/kg) for 15 min prior to heart isolation promotes better recovery of ventricular developed pressure (LVDP) after I/R in the isolated rat heart. The μ OR‐selective antagonist CTAP (0.1 mg/kg) completely abolished DAMGO's protective effect. In contrast, perfusion of the isolated rat heart with DAMGO (0.1 mg/L or 195 nM) for 10 min prior to ischemia did not improve recovery of function.^127^ Only activation of the μ OR in vivo preserves postischemic contractility ex vivo. It is known that *K*_i_ of DAMGO for μ OR is 1.23^110^ or 27 nmol.^210^ Therefore, we cannot explain an absence of inotropic effect of DAMGO ex vivo by a too low concentration of peptide. The protective effect of the μ OR agonist must be dependent upon μ OR activation somewhere outside the heart.^47^, ^64^, ^211^

### B. κ OR versus δ OR

In a study on isolated rat heart, it was seen that perfusion with 200 μM DADLE prior to hypothermic cardiac arrest decreases the postischemic rise in end diastolic pressure (EDP) but not the decline in left ventricular developed pressure (LVDP).^92^ In 1999, Benedict et al. subjected the isolated rabbit heart to cardioplegic arrest (2 h of 34°C ischemia) followed by reperfusion. Perfusion of the isolated heart with morphine prior to ischemia promotes an increase in contractility during reperfusion.^212^, ^213^ The selective μ OR agonist fentanyl^198^ did not have a similar effect. Consequently, it may be concluded that positive inotropic effect of morphine was depended upon δ or κ OR activation. The British physiologists Kato and Foex subjected the isolated perfused rat heart to 30‐min global ischemia and 60‐min reperfusion. The heart was perfused with the μ OR agonist fentanyl (740 nM). Fentanyl increased LVDP, the rate of contraction, and the rate of relaxation of heart during reperfusion and pretreatment with naloxone abolished fentanyl's protective effect. These authors concluded that the inotropic effect of fentanyl was dependent upon δ OR activation.^214^ Kato and Foex gave fentanyl at a concentration sufficient to occupancy of μ, δ, and κ OR.^214^ Therefore, in our opinion, the presented data do not allow one to make a conclusion that the protective effect of fentanyl is mediated via δ OR stimulation. To further complicate the issue, the same authors published a paper in 2000, which reported that there was no improvement of contractility in the reperfusion period after 740 nM fentanyl.^215^ It is unclear, which study is correct.

Exposing the isolated rat heart to the selective κ_1_ OR agonist U50,488 (1 μM) for 2 min starting 10 min prior to global I/R promoted an increase in LVDP in the reperfusion period.^216^ The κ_1_ OR agonist had no effect on the EDP when given only during reperfusion. The inotropic effect of U50,488 was blocked by pretreatment with the selective κ OR antagonist nor‐binaltorphimine (1 μM during 4 min). These authors concluded that the protective effect of U50,488 was dependent upon κ OR activation. Their work could be criticized because they did not use U50,488 and nor‐binaltorphimine at receptor‐selective doses. The *K*_i_ of U50,488 for κ_1_ OR is 0.89 nmol^217^ and the *K*_i_ of nor‐binaltorphimine for κ OR is 0.18 nmol.^218^ Nor‐binaltorphimine at the final concentration of 100 nmol will also inhibit δ OR.^219^ In 2001, Genade et al. found that perfusion of the isolated heart with 10 nmol DADLE prior to ischemia improved mechanical function after reperfusion.^220^ Since DADLE at 10 nmol should only interact with δ OR,^221^ it may be assumed that the inotropic effect of DADLE was dependent upon δ OR activation.

We perfused isolated rabbit heart with 2 mM DADLE for 15 min before cardioplegic arrest and a 2‐h global ischemia followed by reperfusion.^222^ DADLE promoted an increase in LVDP over those hearts that were subjected to only cardioplegia.^222^ DADLE at the concentration of 2 mM activates all ORs.^221^, ^223^ Therefore, it is not clear what OR subtype was involved in the protective effect of DADLE. We continued this study with swine hearts. After pretreatment with DADLE (1 mg/kg intravenously), morphine (1 mg/kg intravenously), or saline, hearts were excised and kept for 75 min at 4⁰C, then reperfused them in a four‐chamber isolated working heart apparatus.^224^ We found that pretreatment with either DADLE or morphine promoted an increase in cardiac output during reperfusion. Since neither DADLE nor morphine are the δ OR‐selective agonists, it remains unclear what OR subtype was involved. We suspect that the protective effect of DADLE was dependent upon μ OR stimulation as noted in another of our studies.^127^ Similar data were obtained by Shinmura et al. They injected the δ OR‐selective agonist BW‐373U86 (1 mg/kg) into rats subcutaneously either for 1‐ or 24‐hr before the heart isolation. Isolated perfused rat hearts were subjected to 20 min of global ischemia followed by 20 min of reperfusion. Pretreatment with BW‐373U86 improved LVDP during reperfusion.^225^ Such evidence indicated that BW‐373U86 mimics both preconditioning and delayed preconditioning. It was not determined as to what OR subtype(s) were involved.

In 2002, Wu et al. subjected isolated perfused rat hearts to 30‐min global ischemia and 2‐h reperfusion. Hearts were perfused with 100 nM [Dmt^1^]DALDA or 1 μM morphine for 30 min and then subjected to 30‐min global ischemia. Reperfusion was performed using the same solutions. Both opioids increased contractile force during reperfusion over that seen with buffer only. The protection was present even when hearts were only perfused with [Dmt^1^]DALDA during reperfusion, whereas reperfusion with morphine only during reperfusion had no effect on the contractility.^226^ It is known that the peptide [Dmt^1^]DALDA is an agonist of μ and κ OR.^227^ Therefore, it remains open as to what OR was involved.

Peart and Gross presented evidence that either δ or κ OR stimulation improves cardiac function during reperfusion.^141^ Isolated murine heart was subjected to 20 min global ischemia followed by 45 min reperfusion. The OR agonists were infused for 10 min prior to ischemia, and then throughout reperfusion. Infusion of 10 μM morphine induced an improvement in postischemic recovery. Infusion with the selective δ OR agonist BW373U86 (1 μM) also improved recovery of LVDP. Pretreatment with the selective δ_1_ OR antagonist BNTX (1 μM) completely abolished this effect of BW373U86. Infusion of the selective κ_1_ OR agonist U50,488 (1 μM) produced a marked improvement in contractile recovery.^141^ This effect was blocked by the selective κ OR antagonist nor‐binaltorphimine (1 μM).

Gross's group showed that pretreatment with the selective δ OR agonist DPDPE (1 μM) also improves mechanical recovery of murine hearts following ischemia.^228^ In another study, cardioplegic arrest during global ischemia (2 hr at 34°C) was induced and followed by reperfusion. Hearts that were pretreated with either the preferential δ OR agonist DADLE or the κ OR agonist U50,488 demonstrated significantly improved functional recovery versus controls. The selective μ OR agonist fentanyl had no effect on recovery.^229^ Selective antagonists were not tested. An improvement of contractility during reperfusion after U50,488 was confirmed in isolated rat hearts.^230^ This effect was abolished after pretreatment with nor‐binaltorphimine indicating the protective effect of U50,488 is mediated via κ OR occupancy. Perfusion of the isolated rat heart with a solution containing the nonselective κ OR agonist pentazocine before or after 15‐min global ischemia improved cardiac contractility during reperfusion.^231^ These authors did not test with the OR antagonists. It was also shown that preliminary perfusion of the isolated heart with 1 μM morphine for 15 min before global ischemia promoted an increase in LVDP in the reperfusion period.^161^ These authors also did not test any OR antagonist. It should be noted that some investigators did not find a positive effect of morphine or U50,488 on cardiac contractility although they did decrease infarct size.^194^, ^232^

## 11. WORSENING OF POSTISCHEMIC MECHANICAL RECOVERY BY OPIOID LIGANDS

In the above studies, we presented data that the OR agonist can prevent an appearance of reperfusion contractile dysfunction. However, there are reports that some opioids can also exacerbate contractile dysfunction. The κ OR agonist bremazocine exacerbates reperfusion contractile dysfunction of the isolated heart.^142^ It is known that bremazocine is a potent κ_2_ OR agonist.^143^ Therefore, the above‐presented data on the effects of the κ_1_ OR agonist U50,488 and bremazocine do not contradict each other.

We observed that intravenous administration of the δ_1_ OR‐selective agonist DPDPE (0.1 or 0.5 mg/kg) 15 min prior to the heart isolation exacerbates reperfusion contractile dysfunction.^112^ If we added 0.1 or 0.5 mg/L DPDPE (154 or 771 nM) 15 min before global ischemia (45 min) and reperfusion (30 min), we also observed exacerbation of contractile dysfunction. DPDPE peptide can interact with only δ_1_ OR at the final concentration of 154 nM.^198^ Pretreatment with the selective δ OR antagonist naltrindole (1 nM) completely abolished the negative inotropic effect of DPDPE (154 nM).^106^ We later found that the selective δ_1_ OR agonist TAN‐67 (178 nM) also exacerbates dysfunction during reperfusion.^103^ Pretreatment with the selective δ OR antagonist naltrindole (1 nM) abolished this effect of TAN‐67. Our above result is drastically different from the data of Gross's group^141^, ^228^ where they generally found protection from δ_1_ OR agonists. It worth mentioning that their schedule of drug administration was quite different from that used in the above studies, however.

In most of the above studies, the ischemic period was long enough to cause some necrosis of the heart. In those studies, the postischemic recovery is influenced by a combination of stunning and infarction; so it is not clear which was contributing to an enhanced postischemic improvement in mechanical function. This is important in that the mechanisms of the two forms of injury differ drastically. In 2006, Grosse Hartlage et al. employed a pure stunning model where a coronary branch of a chronically instrumented dog is given a 10‐min coronary occlusion, which is too short to cause any infarction but does depress postischemic function. Function completely recovers spontaneously in a day proving that the segment was only stunned. They gave the selective κ OR receptor antagonist nor‐binaltorphimine (2.5 mg/kg intravenously). Pretreatment with the κ OR blocker prevented the decrease in ventricular wall function after ischemia. They found evidence that the endogenous opioid dynorphin was elevated in the plasma after the ischemic insult and concluded that this opioid was exacerbating the dysfunction in the untreated dogs.^233^ These data contradict the abovementioned data on positive inotropic effect of the κ OR agonist U50,488 during reperfusion^141^, ^230^ but those studies used isolated hearts with long ischemic periods where the agonist was confined to the pretreatment period. We found that perfusion of the isolated rat heart with solution containing U50,488 (0.1 μM) starting 10 min before global ischemia (45 min) decreases creatine kinase release during reperfusion but depresses the recovery of contractile dysfunction.^120^ If we used U50,488 at the final concentration of 1 μM, the cardioprotective effect disappeared but the negative inotropic effect was enhanced.

Thus, results of studies of the inotropic effects of opioids on cardiac stunning are very contradictory. Some studies indicated that pretreatment with opioids improves cardiac contractility in reperfusion period.^92^, ^127^, ^161^, ^213^, ^214^, ^216^, ^220^, ^222^, ^225^, ^226^, ^230^, ^231^ Other studies showed that pretreatment with opioids exacerbate contractile dysfunction.^103^, ^106^, ^112^, ^120^, ^142^ Other investigators could not find any alteration of postischemic recovery after pretreatment with the OR agonists.^127^, ^194^, ^214^, ^232^ Much of this confusion no doubt arises from heterogeneity in the models (isolated vs. in situ), the schedule of drug administration (pretreatment vs. post treatment vs. continuous treatment), and the type of injury (infarction vs. stunning). Therefore, the resolution of possible inotropic effects of opioids during myocardial reperfusion remains to be determined.

## 12. ANTIARRHYTHMIC EFFECT OF THE OPIOID RECEPTOR LIGANDS

The most frequent causes of death from myocardial infarction are cardiogenic shock (52%), arrhythmias (25%), thromboembolism of the pulmonary artery (10%), and rupture of the left ventricle (5%).^234^ These findings indicate that an antiarrhythmic drug could dramatically reduce mortality in this population. Opioids are potential candidates for developing such drugs.

The first report that an OR agonist has an antiarrhythmic effect was with meptazinol during coronary artery occlusion in rats in 1983.^235^ In 1989, it was shown that the selective μ OR agonist fentanyl (60 μg/kg intravenously) increased the ventricular fibrillation threshold (VFT) in dogs with coronary artery occlusion.^236^ The μ and κ OR agonist buprenorphine had the same effect.^236^ The antifibrillatory activity of the μ OR agonists fentanyl, sufentanil, and carfentanil in dogs with coronary artery occlusion was demonstrated by Hess et al. in 1989.^237^ Clinical observations established that fentanyl (60 μg/kg intravenously) could prevent the appearance of intraoperative ventricular fibrillation during cardiosurgery intervention in neonates.^238^ These studies indicate that opiates can increase cardiac tolerance to the arrhythmogenic effect of I/R.

Unfortunately, these historical studies were performed before highly selective OR antagonists were widely available; so none of these publications contained this approach aimed to confirm a receptor‐mediated effect and identify which subtype was responsible. Morphine is a μ OR‐selective agonist as is fentanyl.^198^ However, it was later shown that the cardioprotective effects of morphine^10^ and fentanyl^214^ are actually dependent upon δ OR stimulation. Furthermore, both narcotic analgesics easily penetrate through the BBB. Therefore, it was unclear whether their antiarrhythmic effect was dependent upon the central or peripheral OR occupancy.

### A. μ OR agonists

In order to find out whether the peripheral ORs are involved in the arrhythmogenesis, we used D‐Ala^2^,Leu^5^,Arg^6^‐enkephalin (dalargin). This compound can penetrate the BBB at a dose of 0.5 mg/kg and higher.^209^ We found that intravenous administration of dalargin (0.1 mg/kg) decreases the incidence of ventricular fibrillation during coronary artery occlusion in rats.^239^ Other investigators confirmed our data in experiments on cats.^240^ According to the data of Grekova et al. dalargin (0.1 mg/kg) exhibits an antiarrhythmic effect when administered intravenously to dogs 5 min prior to coronary artery occlusion.^241^ Dalargin can prevent both ischemic and reperfusion arrhythmias. However, this opioid peptide was ineffective if it was administered after coronary artery ligation.^241^ Dalargin not only prevented the appearance of arrhythmias during ischemia, it also evoked an increase in the VFT in rats with postinfarction fibrosis.^242^ We therefore reasoned that the antiarrhythmic effect of dalargin is mediated via peripheral OR activation. However, it is still unknown what OR subtypes are involved in antiarrhythmic effect of dalargin because this peptide is a μ and δ OR agonist.^243^, ^244^

We found that the injection of the selective μ OR agonist DALDA prior to a 10‐min coronary artery occlusion and reperfusion in rats does not affect the incidence of ventricular arrhythmias.^138^ Nor could we find an antiarrhythmic effect of the selective μ OR agonist DMGO (150 or 1500 nmol/kg) or the μ OR agonist dermorphin H (150 nmol/kg) during coronary artery occlusion.^245^ In experiments with postinfarction cardiac fibrosis we obtained data that the nonselective μ OR agonists morphine and dalargin and the μ OR agonist DALDA increase the threshold for fibrillation.^242^, ^246^, ^247^ The antifibrillatory effect of DALDA (0.1 mg/kg) was not present after inhibition of the peripheral ORs with naloxone methiodide.^247^ We theorize that the antifibrillatory effect of DALDA is depended upon peripheral OR occupancy. Blockade with the μ OR‐selective antagonist CTAP (0.5 mg/kg) also abolished the antifibrillatory effect of DALDA.^247^ Therefore, it can be argued that DALDA induces an increase in cardiac electrical stability via the peripheral μ OR activation. Thus, peripheral μ OR activation increases cardiac electrical stability in animals with postinfarction cardiac fibrosis but, based on our data, μ OR agonists do not suppress acute I/R arrhythmias.

### B. δ OR and Arrhythmias from Acute I/R

In more recent experiments with the selective δ_1_ OR agonists TAN‐67 (0.08 mg/kg), DPDPE (0.1, 0.2 and 0.5 mg/kg), and the selective peptide agonist DSLET (0.11 mg/kg), we found that these ligands after intravenous administration had no effect on the incidence of ventricular arrhythmias during a 10‐min coronary artery occlusion and reperfusion in rats.^138^ However, we found that selective δ_2_ OR activation with intravenous administration of deltorphin II (0.12 mg/kg or 150 nmol/kg) did reduce arrhythmias from I/R.^138^ We suspect that the strong antiarrhythmic effect of deltorphin II and the absence thereof, in DSLET is a consequence of different affinities of these ligands to δ OR. Deltorphin II exceeds DSLET in two‐fold in its affinity for δ OR.^248^ Therefore, it is not surprising that when using both peptides in equimolar doses only deltorphin II was antiarrhythmic. Recently, we have shown that the selective δ_2_ OR agonist deltorphin II (150 nmol/kg), the putative δ_2_‐selective agonist deltorphin D_var_ (150 nmol/kg) and deltorphin E (150 nmol/kg) had antiarrhythmic properties during coronary artery occlusion/reperfusion.^245^ We performed further studies on Deltorphin II, which exerted the most pronounced antiarrhythmic effect. Pretreatment with the nonselective OR antagonist naltrexone (5 mg/kg), the nonselective peripheral OR antagonist naloxone methiodide (5 mg/kg), or the selective δ_2_ OR antagonist naltriben (0.3 mg/kg) completely abolished deltorphin II's antiarrhythmic effect. But pretreatment with the δ_1_‐selective antagonist BNTX (0.7 mg/kg) did not abrogate deltorphin II's antiarrhythmic effect. Therefore, we concluded that peripheral δ_2_ OR stimulation enhances cardiac tolerance to arrhythmogenic impact of ischemia and reperfusion.^245^

We found that intravenous administration of the δ_1_ OR‐selective peptide DPDPE at a dose of 150 and 1500 nmol/kg had no effect on the incidence of ischemic and reperfusion ventricular arrhythmias in rats.^245^ Most of the opioid peptides poorly penetrate the BBB.^23^, ^24^, ^209^ We also tested the δ_1_ OR‐selective agonist TAN‐67 and found that this opioid also was not antiarrhythmogenic.^249^ Therefore, we concluded that peripheral δ_1_ OR agonists are not antiarrhythmogenic. However, Fryer et al. demonstrated an antiarrhythmic effect of TAN‐67 in open‐chest rats.^102^ Unlike our study, they gave TAN‐67 at a dose of 10 mg/kg (125‐fold higher the 0.08 mg/kg dose used by us). The higher dose would have penetrated the BBB. The antiarrhythmic effect of TAN‐67 was no longer seen after selective blocking of δ OR.^102^ We suggest that the antiarrhythmic effect of TAN‐67 is mediated via central OR stimulation. We conclude that peripheral δ_2_ OR activation increases cardiac tolerance to the arrhythmogenic effect of I/R while occupancy of the central δ_1_ OR seems to have the same effect.

### C. δ OR and Arrhythmias from Postinfarction Cardiac Fibrosis

The picture is different with arrhythmias generated by postinfarction cardiac fibrosis. Intravenous administration of the selective **δ**_1_ OR agonist DPDPE (0.1 mg/kg) increased the fibrillation threshold by 36% in rats with postinfarction cardiac fibrosis.^250^ The antifibrillatory effect of DPDPE was lost after blockade of peripheral OR with naloxone methiodide or after selective inhibition of **δ** OR with ICI 174,864. The selective **δ**_2_ OR agonist DSLET (0.5 mg/kg) did not exert any antifibrillatory effect. In this study, we did not test selective **δ**_1_ and **δ**_2_ OR antagonists. Nevertheless, we suspect that the antiarrhythmic effect depended upon peripheral **δ**_1_ OR stimulation.

## 13. CONTROVERSY OVER κ OR AND ARRHYTHMIAS

In 1992, it was found that the selective κ OR agonist U‐50,488 (7.5 mg/kg, intravenously) enhanced cardiac tolerance to the arrhythmias from a 30‐min coronary artery occlusion. Because pretreatment with naloxone (2.5 mg/kg) did not eliminate U‐50,488's effect, these authors concluded that the protection was not dependent upon activation of κ OR.^251^ They proposed that the antiarrhythmic effect was caused by an off‐target blockade of fast Na^+^ channels because after injection of U‐50,488 they observed bradycardia and prolongation of the QRS duration (effects that are typical for I class antiarrhythmic drugs).^251^ However, our results did not concur with these data. They gave U‐50,488 at a dose of 7.5 mg/kg and naloxone was administered at a dose of only 2.5 mg/kg, a dose that is not high enough for inhibition of κ OR.^198^ We found that a lower dose of U‐50,488 (1 mg/kg intravenously) prevents ventricular arrhythmias during coronary artery occlusion and reperfusion.^252^ We believe that the dose of U‐50488 (7.5 mg/kg) used by Pugsley et al. was too high, causing it to exhibit nonreceptor effects.

The above researchers tried to compare antiarrhythmic properties of the κ OR agonist PD 129290 and its R,R (+)‐enantiomer that has a low affinity to κ OR.^253^ Both enantiomers at a dose of 3 mg/kg decreased arrhythmias from a 30‐min coronary artery occlusion. Since, naloxone at a dose of 2.5 mg/kg did not abolish this effect, these authors concluded again that the antiarrhythmic effect of both enantiomers was independent of κ OR. Furthermore, both enantiomers increased the QRS duration and in isolated cardiomyocytes, both compounds did inhibit Na^+^ current.^253^ They again concluded that the antiarrhythmic effect of these opioids is due to blocking Na^+^ channels. In a study with isolated cardiomyocytes, the same research group found that U‐50488 and PD 129290 also inhibit Na^+^ current. The κ OR antagonist itself MR2266 did not produce any change in the Na^+^ or K^+^ currents, nor did it alter the channel blocking properties of U‐50,488.^254^ The electrophysiological effects of U‐50,488 were compared with those of the class Ib antiarrhythmic agent lidocaine in rat heart and the sodium currents expressed in *Xenopus laevis* oocytes by using two‐electrode voltage clamp.^255^ Both U‐50,488H and lidocaine produced a concentration‐dependent tonic block of Na^+^ current but U‐50,488H was approximately fourfold more potent than lidocaine. These authors maintain that the antiarrhythmic properties of the κ OR agonists do not depend on OR activation and is an outcome of nonspecific Na^+^ channel blocking.^253^, ^254^, ^255^

We continued the study using U‐50488 enantiomers, which differ in affinity to κ_1_ OR.^217^ It appeared that (–)‐trans‐(1S,2S)‐U‐50,488 (1 mg/kg intravenously) with high affinity to κ_1_ OR can reduce arrhythmias from 10 min of coronary artery occlusion and reperfusion in rats.^252^, ^256^, ^257^ An enantiomer (+)‐trans‐(1R,2R)‐U‐50,488 with low affinity to κ_1_ OR did not exert similar effect.^252^, ^256^, ^257^ Others have shown that the preferential κ_1_ OR agonist dynorphin A_1‐13_ at a dose of 40 μg/kg intravenously also exerts antiarrhythmic effect in cats with coronary artery occlusion.^258^ Furthermore, we established that pretreatment with the selective κ OR antagonist nor‐binaltorphimine (9 mg/kg, intravenously) completely abolished antiarrhythmic effect of (–)‐U‐50,488.^256^ Pretreatment with the κ_2_ OR antagonist quadazocine (3 mg/kg, intravenously) did not alter antiarrhythmic effect of (–)‐U‐50,488.^256^ The selective κ_2_ OR agonist GR‐89696 (25 μg/kg, intravenously) also had no effect on the reperfusion arrhythmias in rats.^259^ However, inhibition of peripheral ORs with naloxone methiodide (5 mg/kg, intravenously) completely abolished antiarrhythmic effect of (–)‐U‐50,488.^259^

Comparison of the above observations convinced us that peripheral κ_1_ OR activation can enhance cardiac tolerance to I/R‐generated arrhythmias. The basis for this assertion are our data that the selective κ_2_ OR agonist GR‐89696 does not exhibit antiarrhythmic properties and that the κ_2_ OR antagonist quadazocine did not abolish the antiarrhythmic effect of (–)‐U‐50,488.^259^ But pretreatment with naloxone methiodide or nor‐binaltorphimine does abolish the antiarrhythmic effect of (–)‐U‐50,488.^259^ At high concentration, (–)‐U‐50,488 clearly does block sodium channels. But at a low dose, the antiarrhythmic effect of U‐50,488 is mediated via κ_1_ OR occupancy alone.

We determined that intravenous administration of the selective ORL1 agonist nociceptin at a dose of 220 or 1500 nmol/kg had no effect on the incidence of ischemic and reperfusion‐induced ventricular arrhythmias in vivo.^245^ However, nociceptin is not resistant to enzymatic hydrolysis. Therefore, we cannot completely exclude the possibility that a selective ORL1 agonist will exhibit antiarrhythmic properties.

## 14. ARRHYTHMOGENIC AND PROARRHYTHMIC EFFECTS OF OPIOIDS

It has been shown that injection of the nonselective OR agonist β‐endorphin into perfusion solution caused only atrial fibrillation and atrioventricular block in isolated rat heart.^260^, ^261^ It should be noted that in vivo β‐endorphin exhibits antiarrhythmic properties in anesthetized cats with coronary artery occlusion.^262^ In 1987, Lee and Wong demonstrated that injection of the preferential κ OR agonist dynorphin1‐13 (20 μg/heart) caused both atrial and ventricular arrhythmias in isolated rat heart.^263^ This effect was antagonized by naloxone. In 1990, Wong et al. published results of their experiments on isolated Langendorff‐perfused rat heart.^264^ The OR agonists and antagonists were injected directly into the aorta through cannulas. The OR antagonists were administered 1 min before the administration of OR agonist or 20‐min global ischemia and 60‐min reperfusion. The selective μ OR agonist DAMGO evoked atrial arrhythmias and at a higher dose caused frequent premature ventricular contractions (PVC).^264^ The selective κ OR agonist U50488 caused both atrial and ventricular arrhythmias. At a high dose (132 nmol/heart), this opioid induced frequent PVC and ventricular tachycardia. The δ OR agonists DPDPE and DADLE evoked only atrial arrhythmias. The arrhythmogenic effects of U50488 were attenuated by pretreatment with the κ OR antagonist MR 2266 in a dose‐related manner whilst the proarrhythmic effect of DAMGO was abolished by the preferential μ OR antagonist naloxone. Naloxone itself exhibited a weak antiarrhythmic effect manifested in prevention of only ventricular tachycardia during reperfusion. Authors concluded that the cardiac κ OR are the most likely receptors involved in arrhythmogenesis during ischemia and reperfusion.^264^ The limitation of this study was the fact that the bolus administration of opioids did not allow them to compare the concentrations of the opioids with their respective Kds and Kis. Consequently, this work does not allow evaluating the role of OR subtypes in the arrhythmogenic effects of opioids.

MR 2266 also exhibits an antiarrhythmic effect. Therefore, the combined use of MR 2266 and U50488 could eliminate the arrhythmogenic effect of U50488 regardless of OR blockade. Later it was shown that intravenous administration of dynorphin (300 nmol/kg) diminished the arrhythmogenic effect of coronary artery occlusion in rats.^265^ This effect was abolished by pretreatment with naloxone (1 mg/kg). However, since naloxone itself exhibits an antiarrhythmic effect, it remains unclear whether the inhibition of the proarrhythmic effect of dynorphin by naloxone occurred due to the blockade of ORs or the antiarrhythmic effect of naloxone overshadowed the proarrhythmic effect of dynorphin. Interestingly, according to others, dynorphin A1‐13 (25 nmol/kg) prevents the occurrence of ventricular fibrillation in anesthetized cats subjected to occlusion of the left coronary artery.^266^ In 2003, Coles et al. published a comparative study of the cardiovascular effects of opioids in pigs with coronary artery occlusion (45 min) and reperfusion (3 hr).^267^ They found that the preferential δ OR agonist DADLE (1 mg/kg) and the preferential κ OR agonist pentazocine (5 mg/kg) aggravated the arrhythmogenic effect of coronary artery occlusion. The selective κ OR antagonist nor‐binaltorphimine (1.5 mg/kg) exhibited the same effect. However, pretreatment with nor‐binaltorphimine completely abolished the proarrhythmic effect of both DADLE and pentazocine. The authors concluded that κ OR activation during ischemia exhibits proarrhythmic effect in pigs.^267^ Indeed, it is known that DADLE can activate κ ORs in isolated hearts and we cannot exclude the possibility that its proarrhythmic effect is mediated by activation of these receptors.^143^ However, we would like to draw the readers’ attention to the following paradox: the κ OR antagonist nor‐binaltorphimine also had a proarrhythmic effect that disappeared when treatment with the κ OR antagonist was combined with the κ OR agonist during ischemia. It is worth mentioning that the authors used high doses of the OR agonists. Meanwhile it is well known that opioid peptides at high concentration may interact with non‐ORs.^268^, ^269^, ^270^ Therefore, there is a possibility that the toxic effects of DALDA and pentazocine are unrelated to the ORs but mediated via stimulation of other receptors.

It is worth mentioning that we have never observed an arrhythmogenic or proarrhythmic effect of opioids administered intravenously to rats or in the experiments on isolated perfused rat heart. We noticed a proarrhythmic effect of DADLE only in pigs during coronary artery occlusion at a high dose of 1 mg/kg.^101^ However, we do not exclude the possibility that opioids may have arrhythmogenic and proarrhythmic effects associated with the activation of central ORs when used at a high dose. We have established that the κ_1_ OR agonist U‐50488, the κ OR agonist [D‐Ala2]‐Dynorphin A(1‐13) and the preferential κ_2_ OR agonist (–)‐bremazocine administered intracerebroventricularly potentiate the arrhythmogenic effect of intravenous epinephrine.^271^, ^272^ Pretreatment with N‐cholinergic receptor antagonist hexamethonium prevented proarrhythmic effects of the intracerebroventricular administration of U50488 and dynorphin.^271^ In contrast, intravenous administration of the preferential κ_2_ OR agonist (–)‐bremazocine and intraperitoneal injection of the selective κ OR agonist spiradoline blunted the arrhythmogenic impact of epinephrine.^272^ This effect was abolished by pretreatment with nor‐binaltorphimine but not hexamethonium or atropine. These data indicate that stimulation of the central κ OR may promote a proarrhythmic effect mediated by the autonomic nervous system. Stimulation of peripheral κ OR may have an antiarrhythmic effect that is independent of the autonomic regulation of heart rhythm.

Thus, we do not exclude the possibility that high doses of opioids may have proarrhythmic and arrhythmogenic effects in humans and animals associated with the activation of non‐ORs or activation of central κ ORs. There is also a possibility that activation of a cardiac κ OR subtype can also contribute to the appearance of ventricular arrhythmias.

## 15. ANTIARRHYTHMIC ACTIONS OF OPIOID ANTAGONISTS

There are reports that the OR antagonists can also exhibit antiarrhythmic properties during I/R of heart.^273^, ^274^, ^275^, ^276^, ^277^ These investigations showed that intravenous administration of naloxone (1 mg/kg) before coronary artery occlusion in anaesthetized dogs reduced the incidence and severity of cardiac arrhythmias during coronary artery occlusion and reperfusion.^273^ Studies also indicate that pretreatment with the κ_2_ OR antagonist quadazocine (3 mg/kg) or the OR antagonist (–)‐Mr 1452 (4 mg/kg) prevents the appearance of arrhythmias induced by coronary artery occlusion in rats.^274^ These investigators established that naloxone (0.5 mg/kg), the preferential κ OR antagonist Mr 2266 (4 mg/kg), and the nonselective OR antagonist MrZ 2593 (a quarternary complex of naloxone which does not readily cross the BBB at 1 mg/kg) all prevent the appearance of arrhythmias evoked with regional cardiac ischemia in rats.^275^ It has been reported that intravenous administration of the nonselective OR antagonist nalmephene (1 mg/kg) prevents reperfusion‐induced arrhythmias in dogs.^276^ There was also a report that pretreatment with naltrexone (2 mg/kg) or methylnaltrexone (2 mg/kg), a quaternary derivative of naltrexone that does not cross the BBB, prevents ventricular fibrillation induced with coronary artery occlusion in rabbits.^277^

Our studies found that the OR antagonists (naltrexone, naloxone methiodide, naltriben, BNTX, quadazocine, nor‐binaltorphimine, CTAP, β‐funaltrexamine, ICI‐174,864) had no effect on the incidence of ventricular arrhythmias during coronary artery occlusion/reperfusion in rats.^256^, ^257^, ^259^ It is unclear why our data contradict the data of the other researchers. It should be noted that many of the aforementioned studies used antagonists (Mr 1452, Mr 2266, nalmephene, methylnaltrexone) that are no longer used in OR studies because they exhibit OR independent effects. All our studies were performed with rats and perhaps there were also species differences in the response to the OR antagonist. The authors who found an antiarrhythmic effect of naloxone and naltrexone were performed in investigations with dogs and rabbits.^273^, ^277^ The nonselective OR agonist pentazocine reportedly prevented the appearance of ventricular reperfusion arrhythmias in isolated rat hearts.^231^

## 16. THE EFFECT OF COMORBIDITIES ON THE CARDIOVASCULAR EFFECTS OF OPIOIDS

Pathological processes may significantly change the cardiovascular system response to exogenous opioids. For example Bolte et al.^278^ found augmented negative inotropic and lusitropic response to administration of the selective δ OR and κ OR agonists in the failing hamster heart. However, Kasper et al. could not find any difference in the negative inotropic effect of κ OR agonist U‐50,488H on control and cardiomyopathic hamster cardiomyocytes.^279^ The inhibitory action of the selective κ OR agonist U50 488H on β‐adrenoceptor augmentation of voltage‐dependent [Ca^2+^]_i_ transients in the isolated cardiomyocytes appeared to be significantly reduced in spontaneously hypertensive rats.^280^ In 2001, Pei et al. demonstrated that the effect of U50,488H on the [Ca^2+^]_i_ transient in the isolated cardiomyocytes was significantly attenuated due to right ventricular hypertrophy induced by chronic hypoxia.^281^ The authors established that κ OR signaling was impaired in the hypertrophied cardiomyocytes due to a defect in the coupling between κ OR and PKC. It was also found that high fat‐induced obesity alters cardiovascular response to the administration of opioids in conscious rats.^282^

The pathological process itself may change the state of the endogenous opioid system. It has been shown that cardiomyopathy evoked an increase of the preproenkephalin A mRNA level in ventricles of hamsters.^283^ In spontaneously hypertensive rats, the heart content of dynorphin A was increased by 6.5‐fold compared to Wistar rats.^284^ Plasma β‐endorphin levels are also elevated in dogs with pacing‐induced congestive heart failure. Naloxone injection increased HR, mean aortic pressure, first derivative of left ventricular pressure and cardiac output in these dogs while in the intact animals, naloxone did not affect the hemodynamics.^285^ These data suggest the involvement of endogenous opioids in the pathogenesis of pacing‐induced congestive heart failure. This hypothesis can be supported by the data of Imai et al.^286^ They found that not only naloxone but also the selective δ OR antagonist ICI‐154,129 increased mean aortic pressure, cardiac output and positive first derivative of left ventricular pressure in dogs with artificially induced right heart failure. Constriction of the aorta induced an elevation of β‐endorphin level in blood plasma of rats.^287^ In 1995, Oldroyd et al. found that plasma β‐endorphin was increase by 29% in patients with acute heart failure and by 71% in patients with cardiogenic shock.^288^ However, the level of this opioid in plasma was decreased in spontaneously hypertensive hamsters.^289^ It was demonstrated that the decrease in left ventricular systolic pressure after administration of the κ OR agonist U50488H was attenuated in these hamsters.

These results show that pathological process can exert a significant effect on the endogenous opioid system. Can the accompanying pathological process change the cardioprotective effect of opioids? It has been demonstrated that aging does not alter a cardioprotective effect of BW373U86, a selective δ OR agonist.^168^ However, in 2007, Peart et al. reported that the selective δ OR agonist DPDPE only improves contractile recovery after reperfusion of isolated mouse hearts from young animals.^228^ They found that aging‐related loss of δ‐opioid‐mediated cardioprotection involves failure to activate p38 MAPK (mitogen‐activated protein kinase) and HSP27 (heat shock proteins). Gross's group demonstrated that morphine did not limit the infarct size in rats with streptozotocin‐induced diabetes. This lack of protective effect was associated with the loss of coupling between ORs and glycogen synthase kinase 3β (GSK‐3β).^290^ It was also shown that remifentanil reduced myocardial infarct size and prevented apoptosis of cardiomyocytes evoked by I/R in nondiabetic rats but not in rats with streptozotocin‐induced diabetes.^89^ Gross's group's data were confirmed by Chen et al.^199^ They demonstrated that sufentanil reduced myocardial infarct size in the nondiabetic rats, but not those with streptozotocin‐induced diabetes. The GSK‐3β inhibitor SB216763 reduced infarct size in both nondiabetic and diabetic rats. The authors concluded that absence of opioid‐induced tolerance of rat heart to reperfusion injury in diabetic animals is the result of impaired interaction of ORs and GSK‐3β.^199^

The results of these studies cannot be mechanically applied to the humans because the most common form of diabetes in humans is a type 2 diabetes mellitus characterized by insulin resistance. Streptozotocin causes damage to the β cells of Langerhans islets and leads to decreased insulin secretion. Therefore, streptozotocin‐induced diabetes is most similar to insulin‐dependent type 1 diabetes mellitus in humans. However, Tsang et al. showed in experiments using a rat model of type 2 diabetes mellitus (Goto‐Kakizaki rats) that diabetes depresses the PI3K/Akt pathway during IP causing loss of protection. But the elevated threshold for Akt phosphorylation and protection can be reached by simply increasing the number of IP cycles.^291^ Since this same signaling pathway is involved in the protective mechanism of opioids, as we discuss in the Section 18, it seems likely that an increasing the dose of an OR agonist may still be able to protect the diabetic heart against I/R injury. Taken together, the presented data suggest that diabetes and age may undermine the efficacy of opioid‐induced cardioprotective effects. It remains unknown whether atherosclerosis, arterial and pulmonary hypertension, myocardial hypertrophy, or heart failure might also alter their protection. The phenomenon of heart resistance to cardioprotective stimuli due to comorbid diseases is potentially a serious problem in the translation of cardioprotective interventions to clinical practice.^292^ However, this problem may be solved by either increasing the stimulus or by direct activation of the downstream components of the signaling pathways such as p38 MAPK/HSP27 or inhibition of GSK‐3β, which remain relatively intact in these conditions.

## 17. PROSPECTS FOR THE USE OF OPIOID RECEPTOR AGONISTS IN CARDIOLOGICAL PRACTICE

It has been found that the chronic administration of morphine increases cardiac tolerance to ischemia and reperfusion.^293^, ^294^, ^295^, ^296^, ^297^ The signaling mechanism of the cardioprotective effect of chronic morphine administration differs from that of acute administration of morphine.^296^ Chronic κ‐OR stimulation prevents isoprenaline‐induced cardiac hypertrophy and fibrosis.^298^ Unfortunately, morphine and heroin in chronic administration cause rapid formation of drug dependence.^299^ The formation of opioid dependence is associated with activation of central μ OR.^300^ The ability to form a dependence is much less pronounced in the δ OR or κ OR agonists.^300^ Therefore, δ OR and κ OR agonists are not on the DEA‐controlled substances list. However, since the κ OR agonists cause dysphoria, their indication for chronic use is also unlikely.^301^ In our opinion, the most promising agents for chronic use are peptide OR agonists, that poorly penetrate the blood–brain barrier.^22^, ^23^, ^24^ These include the selective μ OR agonist DALDA (NH_2_‐Tyr‐D‐Arg‐Phe‐Lys‐NH_2_)^227^ and nonselective μ OR and δ OR agonist dalargin (H‐Tyr‐D‐Ala‐Gly‐Phe‐Leu‐Arg‐OH).^302^ Our experiments have shown that dalargin exhibits antifibrillatory properties.^239^ In Russia, this drug is used to treat stomach ulcers.^303^ We have shown that a quaternary analogue of the κ opioid agonist U‐50488 is another opioid that does not penetrate the BBB and is capable of increasing cardiac tolerance to I/R.^123^ Therefore, we suggest that those opioids, which are not able to activate the central ORs after systemic administration, might be most amenable to chronic use as cardioprotectants in high‐risk cases.

Based on the data accumulated by our and Gross's group,^129^ the most promising compounds for development of drugs, which acutely increase the heart's tolerance to the detrimental effects of ischemia and reperfusion are deltorphin II, ICI 199,441 and Eribis peptide 94. Following clinical trials, these compounds or their analogs may find use in the treatment of acute myocardial infarction or in surgergical patients receiving coronary bypass.

## 18. SIGNALING MECHANISM OF THE CARDIOPROTECTIVE EFFECT OF OPIOIDS

**Figure 1:**
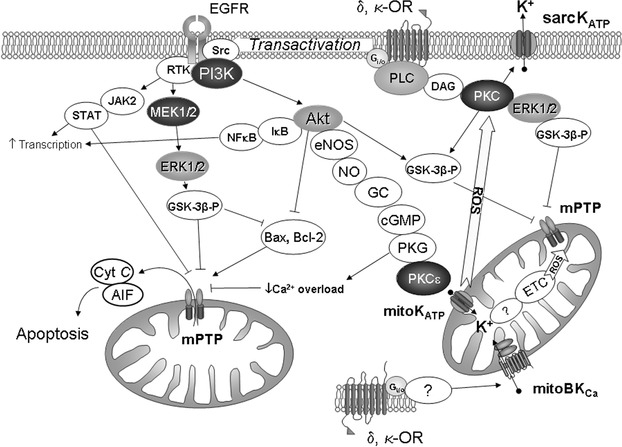
Proposed signaling scheme for the cardioprotective effect of opioids.OR, opioid receptor; PLC, phospholipase C; DAG, diacylglycerol; PKC, protein kinase C; Src, sarcoma tyrosine kinase; PI3K, phosphatidylinositol 3‐kinase; JAK2, Janus kinase 2; STAT, signal transducer and activator of transcription; Akt, kinase isolated from AKR thymoma cell line; MEK1/2, mitogen‐activated protein kinase kinase; ERK1/2, extracellular‐signal‐regulated kinase; NO, nitric oxide; NOS, NO‐synthase; GC, guanylyl cyclase; cGMP, cyclic guanosine monophosphate; PKG, protein kinase G; mitoKATP, mitochondrial ATP‐sensitive K^+^ channel; sarcKATP, sarcolemmal ATP‐sensitive K^+^ channel; GSK‐3β, glycogen synthase kinase‐3β; mPTP, mitochondrial permeability transition pore; EGFR, epidermal grows factor receptor; RTK, receptor tyrosine kinase; Cyt C, cytochrome C; AIF, apoptosis inducing factor; Bcl‐2, B‐cell lymphoma protein‐2; Bax, Bcl‐2‐associated X‐protein; ROS, reactive oxygen species; NFκB, nuclear factor κB; IκB, IκB kinase; mitoBKCa, mitochondrial big conductance Ca2^+^‐dependent K^+^ channel; ETC, electron transport chain.

The data described below are summarized in the signaling scheme for the cardioprotective effect of opioids presented in Figure 1. The phenomenon of IP was found to be triggered by activation of G_i/o_‐coupled adenosine receptors that activate PKC and ultimately protects by inhibiting MPT early in reperfusion.^304^ Soon it was discovered that bradykinin and OR act in parallel with the adenosine receptors as all three are populated during a preconditioning ischemia. Occupation of any one receptor type can put the heart into a conditioned state. It is hypothesized that ORs couple with G_i/o_ proteins, which then inhibit adenylyl cyclase and activate phospholipase C, which, in turn, synthesizes diacylglycerols stimulating protein kinase C.^248^ Therefore, investigators have attempted to determine if the cardioprotective effect of opioids is associated with G_i/o_ protein activation. In a study with rats, it was shown that the G_i/o_ protein inhibitor pertussis toxin eliminated the infarct‐reducing effect of the δ_1_ OR agonist TAN‐67.^91^ Later experiments with isolated perfused murine heart demonstrated that pertussis toxin, which rybosylates the α_i_ subunit of the G_i/o_ protein, eliminated the cardioprotective effect of morphine.^296^ Currently it is hypothesized that G_i/o_ proteins serve as an intermediary link between OR and the protein kinases that perform the protective signaling.

### A. OR Protect through PKC

In 1998, a study with isolated perfused rabbit heart it was shown that the protein kinase C (PKC) inhibitor chelerythrine abolishes the infarct‐sparing effect of morphine.^81^ In the following investigation performed with the isolated perfused rat heart it was found that pretreatment with chelerythrine eliminates the cardioprotective effects evoked by δ and κ_1_ OR in vitro stimulation.^95^, ^105^, ^173^, ^215^

In 2001, Gross's group sought to identify which PKC isoforms are involved in the infarct‐limiting effect of opioids.^305^ They established that the cardioprotective effect of TAN‐67 and DADLE did not occur after inhibition of all PKCs with chelerythrine and after pretreatment with the selective PKC‐δ inhibitor rottlerin. They concluded that the cardioprotective effect of δ OR agonists is mediated via PKC‐δ activation. This result surprised us because PKC δ activation and translocation to mitochondria also promotes cardiomyocyte apoptosis^306^ and inhibition of PKC δ enhances cardiac tolerance to reperfusion injury.^307^ Also preconditioning's protection of adult rabbit cardiomyocytes could be blocked by a PKC ε‐specific antagonist but not one for PKC δ.^308^ However, more recent studies revealed that rottlerin is not a specific PKC‐δ inhibitor.^309^ The key role of PKC in signaling mechanism of antinecrotic effect of opioids was confirmed in later works.^110^, ^296^, ^310^

In a 2001 study, isolated cardiomyocytes were assessed for apoptosis after exposing the cells to hypoxia (12 h) and reoxygenation (12 h). This study indicated that the selective δ agonist BW373U86 prevented apoptosis of cardiomyocytes. Pretreatment with Go‐6976, an inhibitor PKC‐α and PKC‐β, abolished this protective effect of BW373U86.^158^ This study showed the antiapoptotic effect of the δ OR agonist can be attributed to PKC‐α and PKC‐β activation.

A number of studies have demonstrated that PKC is involved in the antinecrotic and antiapoptotic effects of opioids. Debate still revolves around the question of which PKC isoforms are involved in the opioid‐induced enhancement of cardiac tolerance to I/R.

### B. PI3 kinase, Akt, and MAPK—the RISK Pathway

Another set of kinases shown to be in the conditioning pathway are PI3 kinase, Akt, and p42/p44 MAPK. These kinases are collectively called the Reperfusion Injury Survival Kinase (RISK) pathway and are downstream of PKC and are involved in mediating the protection early in reperfusion.^311^ Isolated rabbit cardiomyocytes were subjected to hypoxia and reoxygenation and the endogenous μ and δ OR agonist met‐enkephalin reduced cardiomyocytes death. Pretreatment with the PI3 kinase inhibitor LY‐294002 abolished the cytoprotective effect.^312^ We have found that intravenous U‐50,488 decreases the IS/AAR in rats with coronary artery occlusion and the PI3 kinase inhibitor wortmannin abolished the infarct‐sparing effect.^313^ PI3 kinase phosphorylates membrane phosphoninositide at the 3 position and this product activates phosphoinositide‐dependent kinases (PDKs), which activates Akt by phosporylating it. Recently, it has been shown that the infarct‐limiting effect of sufentanil in vivo also was abolished by pretreatment with wortmannin.^314^

Gross's group found that intravenous administration of morphine (0.3 mg/kg) in rats induces an increase in the phosphorylation of cardiac Akt early in reperfusion.^108^, ^116^ The same group utilizing an isolated murine heart found that DPDPE evoked phosphorylation of Akt.^228^ These data were confirmed by Huang et al.^52^ It was also shown that the phosphorylation of Akt was involved in the cardioprotective effect of remifentanil^162^ and morphine.^315^ Based on the aforementioned studies it appears that PI3 kinase and Akt are involved in cardioprotective effect of opioids.

MAPK comprise a family of kinases involved in growth and cytoprotection. They include p38 MAPK, JNK, p42 MAPK, and p44 MAPK. The last two isoforms are also known as Extracellular Receptor Kinase (ERK). Like all MAPKs, ERK1/2 are activated by two upstream kinase kinases, MEK1/2 which can be inhibited by PD 098059. PD 098059 completely abolishes the anti‐infarct effect of TAN‐67. Furthermore, TAN‐67 increased the phosphorylation of ERKs during reperfusion and this was prevented by PD 098059.^316^ Ikeda and colleagues found that intravenous administration of the δ OR agonist DADLE (1 mg/kg) increased the phosphorylation of both isoforms of ERK in the myocardium.^94^ PD 098059 abolished both ERK's phosphorylation and the protection. van Winkle's group published a study where isolated rabbit cardiomyocytes subjected to hypoxia and reoxygenation were protected with met‐enkephaline and experienced increased phosphorylation of ERK1/2. Pretreatment with PD 098059 or the selective MEK1/2 inhibitor U‐0126 abolished both effects.^312^ The anti‐infarct effect of U50488H could be blocked by an ERK inhibitor but not by one for the PI3K‐Akt pathway.^317^ Ha and colleagues also found that the nonselective OR agonist remifentanil before reperfusion promotes an increase in the phosphorylation of ERK1/2, which could be reversed by naloxone or by a nonselective adenosine receptor inhibitor.^200^ The A2b adenosine receptor has been proposed to be upstream of ERK in the preconditioning pathway.^304^

### C. Other Protective Kinases

In 2011, a study by Li et al. indicated that morphine can ameliorate myocardial contractile dysfunction and limit infarct size following ischemia and reperfusion by a mechanism involving activation of AMPK (AMP‐activated protein kinase).^161^ JNK and P38 MAPK are also members of the MAPK family that have been implicated in cardioprotection. Despite an increase in phosphorylation of both p38 MAPK and JNK by TAN 67, its cardioprotective effect could not be blocked by the p38 MAPK inhibitor SB‐203580.203.^228^, ^305^ Other groups also find an increased phosphorylation of p38 MAPK after morphine use.^189^, ^318^

Morphine induces phosphorylation of GSK‐3β and JAK2.^116^, ^290^ Phosphorylation of GSK‐3β has been proposed to directly inhibit the opening of MPT and is currently regarded as a critical component in the conditioning pathway.^304^ Phosphorylation of GSK‐3β inhibits its kinase activity and the GSK‐3β inhibitor SB‐216763 mimicks morphine's protection. Morphine's protection is lost after inhibition of JAK2. JAK2 is a member of the SAFE pathway and has been implicated in the mechanism of the heart's conditioning phenomenon.^304^ It is unclear whether JAK directly phosphorylates GSK‐3β kinase or acts indirectly through other kinases. DPDPE contributes to phosphorylation p70S6 kinase and GRK2 in isolated rat heart^228^ however, it is not known whether these effects have any relevance to its protective effect.

The endothelial nitric oxide synthase (eNOS) has been implicated in the preconditioning's trigger pathway between G_i/o_ coupled receptors and PKC.^304^ We have found that the infarct‐sparing and antiarrhythmic effects of deltorphin II disappears after pretreatment with the eNOS inhibitor L‐NAME.^117^ We have shown that pretreatment with L‐NAME abolished the infarct‐reducing effect of U‐50,488.^313^ In a study of the cardioprotective effect of Eribis peptide 94 similar data were noted by Gross et al.^129^

### D. Tyrosine Kinases

The kinases discussed above phosphorylate serines or threonines in their target proteins. Another family of kinases only phosphorylate tyrosine residues. Tyrosine kinases are proposed to be involved in the transactivation of the epidermal growth factor (EGF) receptor, a step in the OR's trigger pathway for preconditioning.^304^ A 2001 study indicated that the nonselective tyrosine kinase genistein completely abolished the infarct‐sparing effect of TAN‐67 and DADLE but lavendustin, an inhibitor of Src kinase and the EGF receptor, did not affect the cardioprotective effect of TAN‐67 or DADLE.^305^ These investigators concluded that neither Src kinase nor the EGF receptor are involved in the cardioprotective effect of δ agonists but that some tyrosine kinase is involved. Quite opposite findings were obtained by Cao and colleagues.^312^ They subjected isolated cardiomyocytes to hypoxia and reoxygenation and the Src kinase inhibitor herbimycin A completely abolished the cytoprotective effect of met‐enkephalin. Finally, we have also shown that the infarct‐limiting effect of deltorphin II is maintained after pretreatment with genistein.^117^

Studies have shown that the infarct‐sparing effect of morphine and δ agonist FIT disappeared after pretreatment with AG‐490, an inhibitor of the tyrosine kinase JAK2, but not with the JAK3 inhibitor ZM‐449829.^116^ Morphine induced phosphorylation of JAK2 in the area at risk.^116^, ^290^ It is possible that JAK2 may be the tyrosine kinase that is involved in opioid induced enhancement of cardiac tolerance to I/R.

### E. Interactions between Adenosine Receptors and ORs

Isolated perfused rat hearts were subjected to I/R and fentanyl improved the post ischemic recovery. Naloxone abolished the protection from fentanyl as did the selective adenosine A_1_ receptor antagonist DPCPX.^214^ These authors concluded that the protective effect of fentanyl involved both ORs and the A_1_ receptor. Similar data were obtained by Peart and Gross, they administered either morphine or the selective adenosine A_1_ receptor agonist CCPA to rats subjected to coronary artery occlusion and either compound evoked a decrease in the IS/AAR. The infarct‐limiting effect of morphine was eliminated by DPCPX and after selective blocking of δ_1_ OR with BNTX. Furthermore, the cardioprotective effect of CCPA was blocked after injection of either DPCPX or BNTX.^320^ Coadministration of morphine and CCPA did not offer any additive protection. These authors concluded that there is an interaction between δ_1_ OR and A_1_ receptor at the intracellular signaling level (cross‐talk).

### F. Transactivation of the EGF Receptor

In 2005, Gross's group published data on the cardioprotective effect of endogenous adenosine.^321^ The level of adenosine was elevated with intravenous administration the adenosine kinase inhibitor 5‐iodotubercidin (1 mg/kg). This compound decreased the IS/AAR twofold. Pretreatment with the adenosine A_1_ receptor‐selective antagonist DPCPX, the adenosine A_3_ receptor‐selective antagonist MRS‐1523, or the δ_1_ OR‐selective antagonist BNTX abolished the infarct‐reducing effect of 5‐iodotubercidin.^321^ These data led these researchers to conclude that the cardioprotective effect of endogenous adenosine is mediated via simultaneous A_1_ receptor, A_3_ receptor and δ_1_ OR activation. Recently, Ha et al. found that the nonselective OR agonist remifentanil started 5 min before reperfusion in the isolated rat heart decreases the IS/AAR and evokes the phosphorylation of ERK1/2. These effects were blocked by pretreatment with naloxone or the nonselective adenosine receptor antagonist 8‐(p‐sulfophenyl) theophylline.^200^ It is our opinion that the aforementioned studies provide evidence of interaction of adenosine and OR at some level. It has been proposed that transactivation may be involved as both receptors converge through transactivation of the EGF receptor.^322^, ^323^ However, it is not clear how inhibition of one could turn off signaling of the other through this transactivation.

EGF receptor activation protects against I/R.^312^, ^324^, ^325^ Met‐enkephalin reduces cell death in isolated cardiomyocytes subjected to hypoxia and reoxygenation and phosphorylates the EGF receptor. The EGF tyrosine kinase inhibitor AG‐1478, the Src kinase inhibitor herbimycin A or naloxone eliminated phosphorylation of EGF receptor and the cytoprotective effect.^312^

The aforementioned data indicate that there is an important role of transactivation of opioid and adenosine receptors in cardiac tolerance to the impact of ischemia and reperfusion. Opioid transactivation of EGF receptor tyrosine kinase is a binder link between ORs and ERK1/2 and the downstream signaling pathways including ERK and PI3 kinase. Thus, EGF receptor transactivation is an important mechanism in implementing the protective effect of opioids is now shared by other physiologists.^322^, ^323^

### G. K_ATP_ and BK Channels

Pretreatment with the K_ATP_ channel blocker glibenclamide abolished anti‐infarct effect of morphine.^10^ Two years later, the same group demonstrated that glibenclamide abolished the infarct‐reducing effect of TAN‐67.^91^ It was also shown that the cardioprotective effect of DADLE in vitro disappeared after K_ATP_ channel blockade with this inhibitor.^92^ Further studies were designed to determine what types of K_ATP_ channels are involved in the cardioprotective effect of opioids. It was observed that incubation of cardiomyocytes with morphine prevents cell death and the selective inhibitor of the mitochondrial K_ATP_ channel (mitoK_ATP_) 5‐hydroxydecanoate (5‐HD) completely abolished the cytoprotective effect of morphine.^82^ Pretreatment with fentanyl improved post‐ischemic recovery of function and that was also dependent on the opening of mitoK_ATP_.^214^ In 2000, it was found that the antiarrhythmic and infarct‐sparing effect of TAN‐67 in vivo was abolished by blocking mitoK_ATP_ with 5‐HD but not after blocking sarcolemmal K_ATP_ channels (sarcK_ATP_) with HMR 1098.^102^ The cytoprotective effect of TAN‐67 in isolated cardiomyocytes disappeared after blocking mitoK_ATP_ with 5‐HD.^105^ 5‐HD also blocked the cardioprotective effect of U‐50,488 in isolated perfused rat heart,^326^ abolished the cytoprotective effect of morphine and δ agonist BW373U86 in isolated chicken cardiomyocytes.^107^ In an in vivo study we found that the infarct‐reducing effect of deltorphin II did not occur after blockade of mitoK_ATP_ with 5‐HD.^117^ A large number of similar publications now confirm the key role of mitoK_ATP_ in the cardioprotective effect of opioids.

A few studies also show a role of sarcK_ATP_ in opioid‐induced cardioprotection. The cytoprotective effect of met‐enkephalin disappeared after selective blockade of mitoK_ATP_ with 5‐HD or after selective blockade of sarcK_ATP_ with HMR 1098.^98^, ^99^ The anti‐infarct effect of morphine and BW373U86 in vivo was no longer observed after either mitoK_ATP_ or sarcK_ATP_ blockade.^108^ In another study it was demonstrated that the cardioprotective effect of Eribis peptide 94 was dependent on both mitoK_ATP_ and sarcK_ATP_ opening.^129^

In 2005, it was proposed that the mitochondrial Ca^2+^‐dependent big conductance K^+^ channel (mitoBK_Ca_ channel) was involved in the cardioprotective mechanism of opioids. The κ_1_ OR agonist U‐50,488 provided a decrease in the IS/AAR in isolated rat heart and prevented cell death of isolated myocytes subjected to simulated I/R. The mitoBK_Ca_ channel inhibitor paxilline abolished both effects of U‐50488.^327^

### H. Redox Signaling

The above studies indicate that mitoK_ATP_ and perhaps sarcK_ATP_ are involved in the cardioprotective effect of opioids. It was originally thought that K_ATP_ must be an end effectors of the protection, however, evidence now indicates that their role is primarily one of signal transduction. Studies with preconditioning provided strong evidence that opening of mito KATP activate PKC through redox signaling with free radicals^304^ and that seems to include signaling from the ORs. The anti‐infarct effect of morphine in rabbit heart could be blocked with the free radical scavenger N‐2‐mercaptopropionyl glycine (MPG).^328^ 10 min preconditioning with morphine or BW373U86 increased cell survival of isolated chicken cardiomyocytes subjected to hypoxia and reoxygenation. Morphine‐induced protection and free radical production was abolished by MPG, naloxone, BNTX, a selective δ_1_ OR antagonist; or 5‐HD. The superoxide dismutase inhibitor diethyldithiocarbamic acid exhibited the same effect. Finally, the increase in oxygen radicals was abolished by the mitochondrial electron transport inhibitor myxothiazol.^329^ It was also shown later that the infarct‐sparing effect of morphine does not occur after blocking ROS production with MPG.^320^ In a study with isolated cardiomyocytes subjected to hypoxia/reoxygenation, it was shown that the cytoprotective effect of morphine disappeared after pretreatment with MPG.^315^ Thus, the above data indicate that opioid induced opening of mitoK_ATP_ leads to increased production of ROS, which through redox signaling enhances cardiac tolerance to I/R.

### I. MPT

Elevated cytosolic ROS and Ca^2+^ in the first minutes of reperfusion are thought to open MPT (mitochondrial permeability transition pores).^156^ MPT can destroy the mitochondria and either kill the cardiomyocyte outright (necrosis) or release proapoptotic substances depending on how many mitochondria are lost. A 2005 study indicated that MPT are involved in the cardioprotective effect of opioids.^327^ The regional ischemia and reperfusion was carried out in the isolated perfused rat heart. U‐50488 decreased the IS/AAR and lactate dehydrogenase activity in the coronary effluent of isolated hearts. The MPT opener atractyloside abolished the cardioprotective effect of U‐50488.^327^ It is assumed that OR stimulation inhibited MPT opening at reperfusion and opening MPT directly with atractyloside overrode any inhibition from protective signaling. Mitochondria isolated from the ischemic zone of rat hearts receiving morphine have an elevated threshold for opening MPT with Ca^2+^ and this resistance to MPT opening was lost if PI3 kinase was inhibited with wortmannin.^330^ In our in vivo study we demonstrated that U‐50,488 induces an infarct‐reducing effect as well as an antiapoptotic effect. It also decreases the activity of caspases, which become activated after MPT opening.^163^ The infarct‐sparing and antiapoptotic effects disappeared after blockade of mitoK_ATP_ with 5‐HD. U‐50,488 evoked an enhancement of expression of antiapoptotic protein Bcl‐2 and decreased the expression of proapoptotic protein Bax.^163^ Presumably, the κ_1_ OR agonist prevented MPT opening with signaling through mitoK_ATP_ as discussed above. Kim et al. also found that remifentanil not only limits infarction but also increases Bcl‐2 and decreased Bax.^89^ We conclude that the current evidence indicates that the end effector of the cardioprotective effect of opioids is inhibition of the MPT early in reperfusion.

## 19. WILL AN OR AGONIST PROVIDE PROTECTION TO TODAY'S PATIENT POPULATION?

The ORs appear to protect through a signaling pathway similar if not identical to that of IP and IPost. Evidence suggests that the loading doses of P2Y_12_ receptor inhibitors that are now routinely given to all patients undergoing reperfusion therapy to prevent platelet aggregation in their stents may also trigger this same protection, which could make the OR agonist redundant. The P2Y_12_ blocker cangrelor limited infarct size when present at reperfusion in open‐chest rabbits by an amount similar to that with IPost. Protection from cangrelor could be blocked by 5‐HD, wortmannin, adenosine receptor inhibitors, an ERK inhibitor, or the antioxidant N‐2‐mercaptopropionyl glycine. These same signaling inhibitors will block protection from both IP and IPost. None of those agents restored the ability of platelets to aggregate indicating that protective signaling rather than prevention of thrombi was responsible for the protection. When cangrelor and IPost were combined in rabbits, IPost caused no additional protection, probably because both protect by the same mechanism.^331^

Recent attempts to translate IPost to clinical practice in the setting of acute myocardial infarction have been disappointing. The first IPost trial was performed just before P2Y_12_ blockers came into widespread use and was very positive but all of those performed thereafter had P2Y_12_ blockers present in all patients and showed minimal or no protection from adding IPost.^332^ These data indicate that all of today's patients receiving primary angioplasty to reperfuse their coronary arteries are already in a postconditioned state from their P2Y_12_ blocker loading dose. These drugs were quickly added to the guidelines because they greatly improved clinical outcomes. Their benefit was assumed to result from preventing intracoronary thrombi and the possibility of a direct anti‐infarct effect was not considered. Of course, today it would be impossible to do a clinical trial to measure their ability to reduce infarct size in man because it would be unethical to deny platelet inhibitors to a control group. Any clinically effective agent today must, therefore, be able to provide additional protection when combined with a P2Y_12_ inhibitor. To date, no OR agonists have been tested to see if any of them can provide additional protection in an animal model treated with a P2Y_12_ inhibitor. That screening should be done before considering a large scale clinical trial of any cardioprotectant.

## 20. CONCLUDING REMARKS

Stimulation of central μ OR promotes reduction of infarct size during coronary artery occlusion and reperfusion. Occupancy of peripheral μ OR by opioids promotes better recovery of cardiac contractile function after ischemia. Activation of peripheral and possibly central δ_1_ ORs prevents cardiomyocyte necrosis, apoptosis and arrhythmias caused by I/R of the heart. Stimulation of peripheral OR δ_2_ reduces infarct size and inhibits arrhythmogenesis during coronary artery occlusion and reperfusion. Activation of peripheral κ_1_ ORs also prevents cardiomyocyte necrosis, apoptosis and arrhythmias induced by I/R. Stimulation of peripheral δ and κ ORs contributes to increase in cardiac contractility during reperfusion. No data are available on the involvement of ORL1 receptor in the increased cardiac tolerance to I/R by opioids. It should be noted, however, that at high dose opioids may contribute to cardiac arrhythmias and this effect is probably mediated by non‐OR activation.

The data show that the μ, δ_1_, δ_2_, and κ_1_ OR agonists all are promising candidates for a drug, which would enhance the cardiac tolerance to I/R. The OR agonists exert infarct‐reducing effects both with prophylactic administration and with acute treatment just prior to reperfusion. Furthermore, opioids are also effective in preventing ischemia‐induced arrhythmias.
